# On the role of artificial intelligence in medical imaging of COVID-19

**DOI:** 10.1016/j.patter.2021.100330

**Published:** 2021-08-13

**Authors:** Jannis Born, David Beymer, Deepta Rajan, Adam Coy, Vandana V. Mukherjee, Matteo Manica, Prasanth Prasanna, Deddeh Ballah, Michal Guindy, Dorith Shaham, Pallav L. Shah, Emmanouil Karteris, Jan L. Robertus, Maria Gabrani, Michal Rosen-Zvi

## Main text

(Patterns *2*, 100269-1–100269-18; June 11, 2021)

Due to an error in production, the references for this article were incorrectly numbered upon publication of the typeset paper, May 26, 2021. Since this is a meta-analysis, incorrect numbering of references may have caused misinterpretation of the conclusions. The corrected version of the article is available online as of June 5, 2021, and we sincerely apologize to our readers for any confusion that may have resulted from this error.

