# Supplementary material for: On the role of artificial intelligence in medical imaging of COVID-19
Source: Patterns (N Y). 2021 Apr 30;2(6):100269. doi: 10.1016/j.patter.2021.100269 (PMC8086827; doi:10.1016/j.patter.2021.100269)
Supplement: Document S1. Supplemental appendices [file mmc1.pdf]

Patterns, Volume *2*

Supplemental information

On the role of artificial intelligence in medical imaging of COVID-19

Jannis Born, David Beymer, Deepta Rajan, Adam Coy, Vandana V. Mukherjee, Matteo Manica, Prasanth Prasanna, Deddeh Ballah, Michal Guindy, Dorith Shaham, Pallav L. Shah, Emmanouil Karteris, Jan L. Robertus, Maria Gabrani, and Michal Rosen-Zvi

Appendices

Appendix A. Details on meta-review

Workflow of manual review.
Fig. 1A (main manuscript) shows how the set of 465 papers for manual review was created. First, a search of the keywords "AI + Medical imaging + COVID-19" revealed 827 matches across the 4 servers *PubMed, arXiv, biorXiv and medRxiv*. Those papers were not specific to lung imaging and thus, a second set of keyword searches were made using "AI + Lung + Imaging + COVID-19 + Modality" where Modality was from {CT, X-Ray, Ultrasound}. This revealed 629 publications (visualized in Fig. 2B). Duplicates were removed and subsequently, the titles were scanned manually and papers that only touched peripherally on AI (or one of the other aspects) were removed. This led to a set of 524 papers. From the 524 manually reviewed papers, 61 were excluded during in-depth analysis since they did not involve any work on AI, leading to the final set of 463 papers.

The manual review was split across 7 authors of this paper (AC, DB, DR, EK, JB, MG, VM) and every paper was evaluated by: primary location of authors, primary location of COVID-19 data, imaging modality, task performed, data origin (external or internal) and maturity assessment.

Software for keyword search.
The number of publications per keyword were fetched via a Python package that can be used to reproduce the figures and is publicly available at: https://pypi.org/project/paperscraper/. The queries to the APIs of *PubMed*, *arXiv*, *bioRxiv* and *medRxiv* were made using synonyms or each keyword where a paper was considered a match when title or abstract contained at least one of the synonyms for each keyword (see Table A1). The reference date for all calculations was 31.12.2020.

Table A1 – List of considered synonyms per search keyword.

Keyword	Synonyms
**COVID-19**	**SARS-CoV-2, corona**
**Imaging**	**Image, screen, screening, scan**
**Medical imaging**	**Medical image**
**AI**	**Artificial intelligence, deep learning, machine learning, neural network, computer vision**
**Lung**	**Chest, pulmonary**
**Breast**	**Mammography**
**CT**	**Computed tomography**
**X-Ray**	**XRay, CXR, radiography**
**Ultrasound**	**Sonography, LUS**

Distributions of modality, task and maturity

Figure A1 provides details from the meta-review and quantifies how task and maturity are distributed by modality. For example, 87% of all 230 CXR works performed diagnosis whereas this was only the case for 58% of CT papers, where a richer set of papers was found (~15% of works on segmentation and severity assessment, whereas this was 3-4% for CXR). Projects using X-Ray for diagnosis were on average of much lower quality (82% low quality) compared to CT (60%).

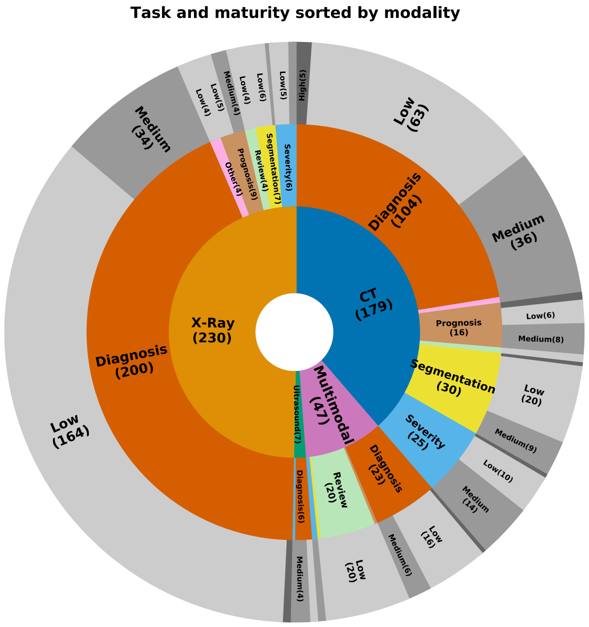

Fig. A1. Sunburst plot on the quality of AI papers distributed by task and modality. The root is in the center and layers are added hierarchically towards the periphery. Hence, the plot should be read inside-out. E.g., From the 179 papers on CT imaging, 104 were dedicated to diagnosis with 63 having low, 36 middle and 5 high maturity. Labels are omitted for fields with less than 5 publications.

Appendix B. Imaging market sizes

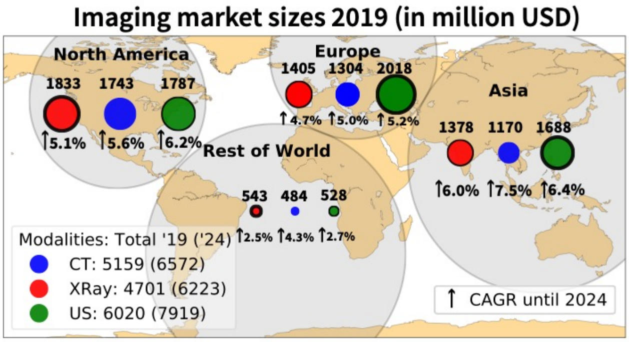

Fig. A2: Regional sizes of medical imaging markets. Overall, US has the largest market across all modalities and is especially dominant in Europe. CT instead is dominant in North America, but CAGR for US are higher and a turn-around is predicted until 2024. The circle radius is proportional to the market size, but for visual clarity, thickness of black borders resembles the regional rank per modality. Figure was created with data from https://www.marketsandmarkets.com/, Diagnostic Imaging Market – Forecast to 2024.

Appendix C. Scheme for maturity assessment

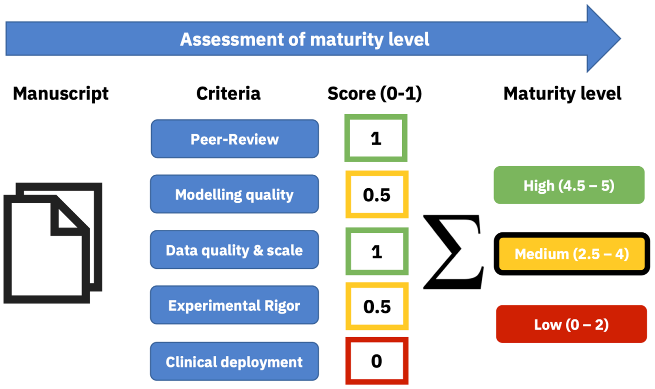

Fig. A3. Workflow for the assessment of maturity of each paper. Each paper was assigned a score for each of the five categories. For details on the individual categories, please see the text.

Criteria

Peer review

Whether or not the article appeared in a peer-reviewed journal or in Conference Proceedings. No distinction between quality of journals/conferences. This is binary score.

Examples:

- **0** Manuscript was not peer-reviewed and is preprint-only (arXiv, bioRxiv, medRxiv)
- **1** Manuscript appeared in a journal or in conference proceedings

Modeling quality

This is an umbrella category for everything related to the AI modelling approach. Mostly, this evaluates how complex/sophisticated the **methods** section of the manuscript is. Did the authors address a single task (e.g., detection) or multiple? Did the authors use off-the-shelf methods or is there clear scientific novelty? Did they devise something specific to their problem? How complex is the problem (e.g., did they only consider imaging data or also clinical tabular data).
The second important factor here is **performance**, i.e., model generalisation (how well does it work?). This should be considered in light of the experimental rigor category. This is a ternary score.

Examples:

- **0** A pre-trained CNN was finetuned on COVID-19 data
- **0** Authors used a proprietary AI software instead of writing their own code.
- **0.5** Some scientific novelty is present but not enough to warrant a “1”. E.g., A small tweak was done to an existing architecture.
- **0.5** Little to no methodological novelty, but the achieved performance is high, the study seems sound.
- **1** A novel and tailored technique was devised to deal with a common problem in imaging and is shown to perform well; ideally on different datasets
- **1** Authors combined several modules for separate problems in their data (e.g., a COVID-19 detection module, a severity assessment module and a module to process clinical features).

Data quality and scale

Quality: The most important sign for high data quality is to have clinical data (as opposed to public). Secondary factors are to have multi-hospital, multi-national, multi-imaging (or generally, multimodal) data.
Scale: How much data is used for training and evaluation. Were multiple datasets used? This is a ternary score.

Examples:

- **0** Only public databases are utilized.
- **0** Authors present a dataset collected from web sources
- **0.5** Authors use several (>3) large public databases and made a significant effort to combine them in a meaningful way.
- **0.5** Authors use public databases as well as clinical data released by other publications.
- **1** Authors present a single-center dataset with >200 patients.

Experimental rigor

This refers to the number of experiments conducted in the Results section. How much results are presented which show different analysis? Did the authors perform significance tests and cross-validation? How about ablation studies? Did the evaluate on multiple, independent datasets? This is a ternary score.
Examples:

- **0** Authors reported performances from single (or repeated) runs on a single data split
- **0.5** Anything that falls in-between of the 0 and 1 descriptions
- **1** Authors compared many different architectures on different datasets and performed cross validation while reporting test data results.
- **1** Authors have few model flavors and single-split but compare them rigorously with statistical tests and ideally even radiologists.

Clinical deployment

Mature AI solution should be deployed in clinical practice or at least evaluated for their prospective clinical applicability in a realistic setting. We decide to be lenient in our interpretation of *clinical deployment.* It describes to what extent the methodology is integrated into (or was evaluated in) a clinical workflow. This is a ternary score.

Examples:

- **0** No mentioning of deployment or comparison with human experts
- **0.5** AI model deployed, but not clinical: Authors built a freely available webservice.
- **0.5** A small human-in-the-loop studies was conducted where human experts interact/compete with the model
- **1** A complex comparison study between the AI and human experts was done
- **1** The AI model was deployed and used in clinical workflow, e.g., to support decision making.
