# Supplementary material for: On the role of artificial intelligence in medical imaging of COVID-19
Source: Patterns (N Y). 2021 Apr 30;2(6):100269. doi: 10.1016/j.patter.2021.100269 (PMC8086827; doi:10.1016/j.patter.2021.100269)
Supplement: Document S2. Article plus supplemental information [file mmc3.pdf]

Patterns

CellPress
OPEN ACCESS

Review

On the role of artificial intelligence in medical imaging of COVID-19

Jannis Born,[1,2,*] David Beymer,[3,*] Deepta Rajan,[3] Adam Coy,[3,4] Vandana V. Mukherjee,[3] Matteo Manica,[1] Prasanth Prasanna,[5,3] Deddeh Ballah,[6,3] Michal Guindy,[7,8] Dorith Shaham,[9] Pallav L. Shah,[10,11,12] Emmanouil Karteris,[13] Jan L. Robertus,[10,12] Maria Gabrani,[1] and Michal Rosen-Zvi[14,15]
[1]IBM Research Europe, Zurich, Switzerland
[2]Department for Biosystems Science & Engineering, ETH Zurich, Zurich, Switzerland
[3]IBM Almaden Research Center, San Jose, CA, USA
[4]Vision Radiology, Dallas, TX, USA
[5]Department of Radiology and Imaging Sciences, University of Utah Health Sciences Center, Salt Lake City, UT, USA
[6]Department of Radiology, Seton Medical Center, Daly City, CA, USA
[7]Assuta Medical Centres Radiology, Tel-Aviv, Israel
[8]Ben-Gurion University Medical School, Be'er Sheva, Israel
[9]Department of Radiology, Hadassah-Hebrew University Medical Center, Faculty of Medicine, Hebrew University of Jerusalem, Jerusalem, Israel
[10]Royal Brompton and Harefield Hospitals, Guy's and St Thomas' NHS Foundation Trust, London, UK
[11]Chelsea & Westminster Hospital, London, UK
[12]National Heart & Lung Institute, Imperial College London, London, UK
[13]College of Health, Medicine and Life Sciences, Brunel University London, London, UK
[14]IBM Research Haifa, Haifa, Israel
[15]Faculty of Medicine, The Hebrew University of Jerusalem, Jerusalem, Israel
*Correspondence: jab@zurich.ibm.com (J.B.), beymer@us.ibm.com (D.B.)
https://doi.org/10.1016/j.patter.2021.100269

THE BIGGER PICTURE During the COVID-19 pandemic, medical imaging (CT, X-ray, ultrasound) has played a key role in addressing the magnified need for speed, low cost, ubiquity, and precision in patient care. The contemporary digitization of medicine and rise of artificial intelligence (AI) induce a quantum leap in medical imaging: AI has proven equipollent to healthcare professionals across a diverse range of tasks, and hopes are high that AI can save time and cost and increase coverage by advancing rapid patient stratification and empowering clinicians.

This review bridges medical imaging and AI in the context of COVID-19 and conducts the largest systematic review of the literature in the field. We identify several gaps and evidence significant disparities between clinicians and AI experts and foresee a need for improved, interdisciplinary collaboration to develop robust AI solutions that can be deployed in clinical practice.

The key challenges on that roadmap are discussed alongside recommended solutions.

SUMMARY

Although a plethora of research articles on AI methods on COVID-19 medical imaging are published, their clinical value remains unclear. We conducted the largest systematic review of the literature addressing the utility of AI in imaging for COVID-19 patient care. By keyword searches on PubMed and preprint servers throughout 2020, we identified 463 manuscripts and performed a systematic meta-analysis to assess their technical merit and clinical relevance. Our analysis evidences a significant disparity between clinical and AI communities, in the focus on both imaging modalities (AI experts neglected CT and ultrasound, favoring X-ray) and performed tasks (71.9% of AI papers centered on diagnosis). The vast majority of manuscripts were found to be deficient regarding potential use in clinical practice, but 2.7% (n = 12) publications were assigned a high maturity level and are summarized in greater detail. We provide an itemized discussion of the challenges in developing clinically relevant AI solutions with recommendations and remedies.

This is an open access article under the CC BY license (http://creativecommons.org/licenses/by/4.0/).

INTRODUCTION

The COVID-19 pandemic has created a desperate need for fast, ubiquitous, accurate, and low-cost tests, and lung imaging is a key complementary tool in the diagnosis and management of COVID-19.[1,2] According to the American College of Radiology (ACR) and the Fleischner Society Consensus Statement, imaging of COVID-19 is indicated in case of worsening respiratory symptoms, and, in a resource-constrained environment, for triage of patients with moderate to severe clinical features and a high probability of disease.[3,4] This involves two main tasks. The first is diagnosis, including incidental diagnosis and providing support evidence in clinical situations in which a false-negative RT-PCR test is suspected. The second task is to help evaluate treatment outcomes, disease progression, and anticipated prognosis. The field of artificial intelligence (AI) in medical imaging (MI) is growing in the context of COVID-19,[5–7] and hopes are high that AI can support clinicians and radiologists on these tasks. In this paper, we review the current progress in the development of AI technologies for MI to assist in addressing the COVID-19 pandemic, discuss how AI meets the identified gaps, and share observations regarding the maturity and clinical relevancy of these developments.

State of artificial intelligence in radiology

Radiologists play a crucial role in interpreting medical images for the diagnosis and prognosis of disease. Although AI technologies have recently demonstrated performance that matches radiologists' accuracy in a number of specific tasks, it remains unclear whether radiologists who adopt AI assistance will replace those who do not. As Celi et al. put it in 2019, "the question is not whether computers can outperform human in specific tasks, but how humanity will embrace and adopt these capabilities into the practice of medicine."[8] A stepping stone toward this long-term vision, however, is the development of AI models that can compete with humans on specific tasks, and a pioneer in that progress is the tremendous success in using AI for detection of breast cancer in screening mammography[9–12]—a success reported by multiple research groups, achieved after 10 years of effort and crowned by OPTIMAM, a database with a total cohort of >150,000 clients.[13]

Similarly, up to 2020, significant progress has been made in diagnosing lung conditions using chest X-ray (CXR) and computed tomography (CT), driven by access to publicized annotated datasets. For example, deep learning (DL)-based approaches outperform radiologists in detecting several pulmonary conditions from CXR[14] and malignancy of lung nodules in low-dose CT.[15] Recently, technologies aiming to assist radiologists in such tasks have been made available on the market.[16] However, several key challenges limit the feasibility of adopting these solutions in practice, namely: (1) poor model generalization due to systemic biases; (2) lack of model interpretability; and (3) non-scalable image annotation processes. Interestingly, similar observations were revealed in the study at hand.

Motivation and contributions

The recent acceleration of publications intersecting AI and imaging for COVID-19 brings a need for rigorous comparative evaluation of papers to summarize and highlight trends to a broad clinical audience. Previous review papers on COVID-19 either focused on a technical assessment of AI in imaging[6] or elaborated on the role of imaging.[1] Related systematic reviews were either not devoted specifically to imaging[17,18] or used extremely small sample sizes (N = 11).[19] In contrast, this paper attempts to bridge clinical and technical perspectives by providing a comprehensive overview to guide researchers toward working on the most pressing problems in automating lung image analysis for COVID-19. Most related to our work, Roberts et al.[20] very recently conducted a systematic review of 62 studies and claim that none of the models are of potential clinical use. While the objective of our work is similar and we also find that the vast majority of manuscripts suffer from methodological flaws, we identify 12 publications that meet substantially higher standards than the remaining manuscripts. Moreover, this work is less focused on assessing individual contributions and more on extracting current trends in the field in a rigorous and comprehensive manner.

Overall, we herein provide the largest systematic meta-analysis of AI in MI of COVID-19 to date. Manually analyzing 463 publications throughout all of 2020 (Figure 1), we attempt to draw a cohesive picture on the current efforts in the field and highlight future challenges, especially related to the cooperation of clinicians and AI experts. While we focus on the lung as the primary organ of SARS-CoV-2 infection, we note the significance of extrapulmonary manifestations.[21]

RESULTS

Progress in AI for medical imaging

In recent years, AI solutions have shown to be capable of assisting radiologists and clinicians in detecting diseases, assessing severity, automatically localizing and quantifying disease features, or providing an automated assessment of disease prognosis. AI for MI has received extraordinary attention in 2020, as attested by a multitude of interdisciplinary projects attempting to blend AI technologies with knowledge from MI in order to combat COVID-19. A keyword search combining AI and MI revealed 2,563 papers in 2019, while 2020 has seen more than twice the number of such papers (5,401, cf. Figure 2). Of these publications, 827 are related to COVID-19, indicating that COVID-19 has accelerated the development of AI in MI.

Lung and breast imaging comparison

To enable a perspective on the emergence of AI for MI of COVID-19, we have compiled a comparison of the progress of automatic analysis in breast and lung imaging, as defined in the literature above, from between 2017 and 2020. Figure 3 (left) shows a stable growth of papers in AI on both lung and breast imaging over the years 2017–2019. In 2020, the rise of lung-related papers has been accelerated by COVID-19 with a doubling in the first half of 2020 compared with the second half of 2019 as well as a doubling of 2020 compared with 2019, whereas the trend on AI on mammography imaging remained unaltered compared with previous years.

Lung imaging modality comparison

To compare the impact of individual modalities, Figure 3 (right) shows that 2019 witnessed a stable trend of ~100–120 papers per quarter on AI whereas with the COVID-19 outbreak in 2020, numbers soared to 164, 352, 372, and 405 papers for

CellPress
OPEN ACCESS

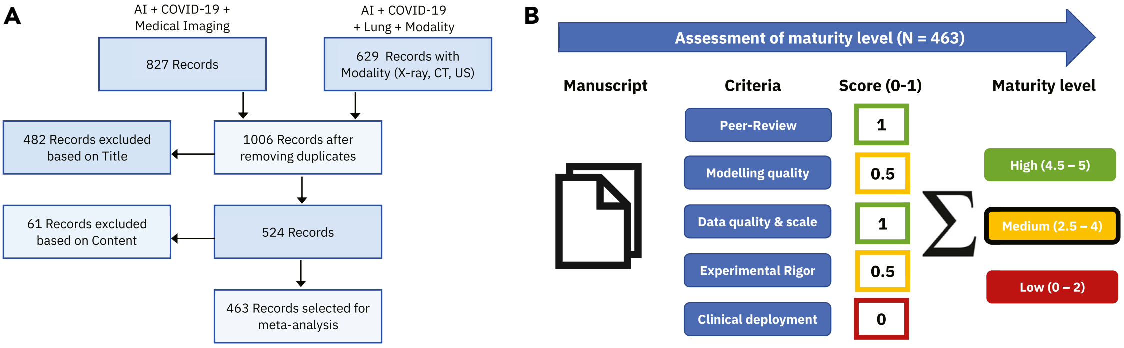

Figure 1. Overview of systematic review and meta-analysis

(A) PRISMA flowchart illustrating the study selection used in the systematic review. Publication keyword searches on PubMed, arXiv, biorXiv, and medRxiv for all of 2020 were performed using two parallel streams. After duplicate matches were removed, titles were screened manually and a selection of 463 relevant manuscripts was chosen for manual review.

(B) Flowchart for quality/maturity assessment of papers. Each manuscript received a score of between 0 and 1 for five categories. Based on the total grade, a low, medium, or high maturity level was assigned. Details on the scoring system and scores for individual papers can be found in supplemental information.

quarter 1 (Q1) to Q4 in 2020, respectively. This rise was spontaneously evoked by COVID-19, as excluding papers mentioning COVID-19 would have resulted in a continuation of the stable trend (see lightly shaded bars) of a hypothetical ~120–160 publications. Notably, the relative contributions of the modalities changed toward CXR from 2019 to 2020 (shares of 71% versus 63% for CT, 27%–35% for CXR, and 2% for ultrasound, respectively). Moreover, for non-COVID-19 papers, the ratio between preprints and PubMed indexed papers for AI in breast and chest is 29% and 37% from 2017 to 2019, respectively; for COVID-19 related papers, this ratio rose to 58%.

Broad insights from meta-analysis

By focusing on CT, CXR, and ultrasound, we quantified the publication efforts of AI for MI of COVID-19 and identified 463 papers, which were included in a manual meta-analysis to review the maturity of the AI technologies and the trends in the rapidly evolving field. The full spreadsheet with individual scores for each publication is available in supplemental information.

Disparity between clinical and AI communities

Of the 4,977 papers about MI and COVID-19 (see Figure 2, right), 2,496 are specific to modalities as shown in Figure 4 (left), indicating a dominance of CT in clinical papers (84%), followed by CXR (10%) and lung ultrasound (LUS) (6%). By using publication counts as an indirect indicator of scientific response, we observe a mismatch in the focus of the AI community in comparison with the clinical community, as illustrated by the distribution of papers per modality in Figure 4 (right) that shows a clear dominance of CXR (50%) across AI papers.

In addition, the vast majority (72%) of papers focused on diagnosis of COVID-19 over tasks such as severity and prognosis (Figure 5, left). This trend is in contrast to the ACR guidelines appraising imaging as an inconclusive test for COVID-19 detection due to uncertainties in accuracy and risk of cross-contamination. Revealing was the unanimous use of CXR data (50%, see Figure 4, right) that was commonly utilized without any further clinical or radiomic features. The tendency for diagnosis was especially prominent for CXR versus CT where 87% and 58% diagnosis papers were found, respectively (cf. the sunburst plot showing task and maturity as distributed by modality in supplemental information, Figure A1). While 6% of papers (27 of all 437 non-review papers) exploited multimodal imaging data toward building their AI models, studies on multimodal imaging data of the same patient cohort are lacking with few exceptions. In one example manual disease airspace segmentation from CT was used as ground truth for volumetric quantification from CXR.[22] Another study demonstrated the diagnostic accuracy of AI on CT to be clearly superior to CXR.[23]

Most AI solutions for COVID-19 have low maturity

The maturity of the papers was assessed following the scheme in Figure 1 (right) by co-authors who have developed or worked with DL algorithms (see Figure 5, middle). Almost 70% of papers were assigned a low maturity level and only 12 (2.7%) highly mature studies were identified. A detailed spreadsheet with the evaluations of each paper is included in supplemental information.

CT papers had a higher maturity score than CXR papers (2.1 ± 1.3 versus 1.3 ± 1.1, $p < 1 \times 10^{-11}$, Mann-Whitney U test) and 57% of CT versus 43% of CXR papers were peer-reviewed. As the pandemic continues the preprint ratio is declining steadily (from 69% in Q1 to 45% in Q4) but not (yet) significantly ($r = -0.93$, $p = 0.07$). The maturity score also heavily varies across performed task and was significantly higher for COVID-19 severity assessment and prognosis (2.4 and 2.5) compared with diagnosis/detection (1.5) and segmentation (1.6) as assessed by Tukey's post hoc HSD multiple comparison tests (Figure 6).

A posteriori, we observed that the monthly citation rate was significantly greater for (1) high compared with medium maturity papers (6.9 versus 2.3, $p < 0.01$, U test) and (2) medium compared with low maturity (2.3 versus 1.9, $p < 0.05$, U test). The continuous maturity score was found to be significantly correlated ($r = 0.12$, $p < 0.05$) with the monthly citation rate. Interestingly however, a major factor accounting for a high citation rate is not the maturity but the months elapsed since publication ($r = 0.35$, $p < 1 \times 10^{-14}$). This suggests that absolute citations and relative citation rates are insufficient quality measures, and we instead observe a tendency toward continuous citation of publications that appeared early in the pandemic (irrespective of their quality).

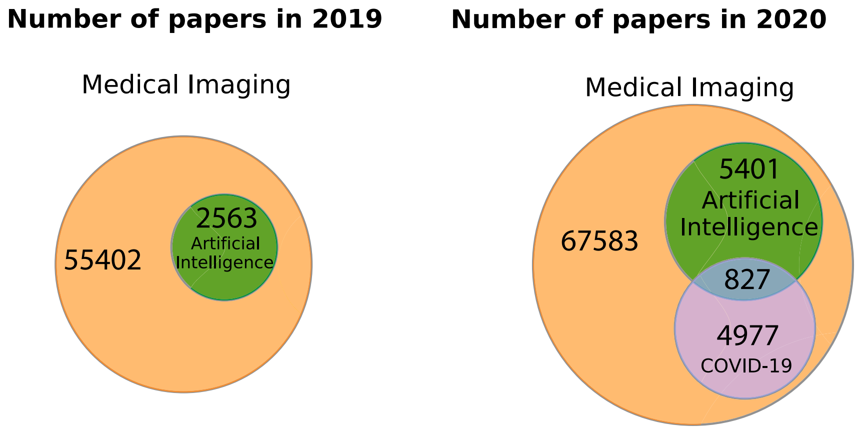

Figure 2. Venn diagrams for AI in MI
MI received growing attention in 2020, at least partially due to the COVID-19 pandemic. Automatic keyword searches on PubMed and preprint servers revealed that AI has been a majorly growing subfield of MI and that 827 publications in 2020 mentioned the terms MI, AI, and COVID-19.

Overuse of small incomprehensive public datasets

We observed that only 30% of papers used proprietary or clinical data (Figure 5, right) while almost 70% analyzed publicly available databases. Such databases exist for CT,[24] CXR,[25] and LUS[26] and are usually assembled by AI researchers, contain data fetched from publications, and comprise no more than a few hundred patients from heterogeneous sources/devices without detailed patient information. Accordingly, the geographical diversity of data sources was not extremely high (26 countries), and by a wide margin the three most important data donators were countries hit early by the pandemic, namely, China (48%) and, to a lesser extent, the United States (12%) and Italy (11%). Interestingly, a global collaborative spirit toward combating COVID-19 was revealed as first-authors from 53 countries and 6 continents contributed to the research, with the most active countries being China (21%), the United States (13%), and India (11%).

Uncovering trends in AI solutions from the mature papers

Twelve (2.7%) of the assessed papers were assigned high maturity.[23,27–37] The list of papers together with details about their task, key findings, implementation, and results appear in Table 1 and are further discussed in this section.

We summarize the trends observed in the identified list of mature papers with a deeper focus on aspects such as (1) choice of AI model architecture, (2) diversity in data sources, (3) choice of evaluation metrics, (4) model generalization, and (5) reproducibility. Furthermore, we highlight common limitations reported in these papers.

1. *AI modeling*. Most of the presented AI solutions have high complexity comprising of multiple modeling stages with at least two models and at most an ensemble of 20 models[35] being trained. Solutions for segmentation tasks tend to model three-dimensional (3D) data, while classification tasks used two-dimensional (2D) data. Almost all of the solutions used transfer learning with pretraining on ImageNet or other open-source clinical datasets (e.g., CheXpert, COPDGene). Popular neural network architectures used included UNet, ResNet, DenseNet, and InceptionNet.
2. *Data sources.* The majority of mature publications utilized data obtained from multiple hospitals containing imaging data from about 500–5,000 patients. The datasets were typically labeled using manual annotations from radiologists, RT-PCR tests, and results from radiology reports. Note that only three studies utilized clinical metadata in addition to images to develop their AI system.[27–29]
3. *Evaluation metrics.* The publications addressing diagnosis tasks commonly used metrics such as accuracy, AUC, sensitivity, and specificity to evaluate the model performance, while using Dice and intersection over union scores to quantify performance on segmentation tasks. The Pearson correlation coefficient was routinely used to compare model and human reader performances and understand the influence of learned features on the overall system performance.
4. *Experimental rigor and model generalization*. We observed that while most publications reported confidence intervals and performed statistical tests, they evaluated their algorithm typically only on a single random split of the dataset. Most mature publications reported model performance on external test datasets, as well as presented heatmaps to illustrate regions of image the model focused on. However, few conducted cross-validation and ablation studies to understand the generalization capabilities of their models. Furthermore, a couple of solutions were deployed in clinical practice[31,36] while another was also thoroughly tested in multiple countries.[27]
5. *Reproducibility*. All of the mature publications used a human-in-the-loop (about 1–8 experienced radiologists) to compare and evaluate their proposed AI solutions, thus making such an evaluation scheme a standard practice. Moreover, a majority of the studies released the code for their algorithm publicly, while the data usually remained proprietary, but was at least partly released in four mature papers.[27,29,30,32]
6. *Limitations*. All publications acknowledge limitations in their studies owing to inherent biases that are modeled into in the datasets through limited size, lack of diversity, and imbalance in disease conditions. In many situations, the datasets represented population of patients with higher prevalence of COVID-19 at the time of imaging, which does not reflect true disease prevalence. Furthermore, the models were deemed sensitive to motion artifacts and other subtypes of lesions or comorbidities that cause data distribution shifts. Most studies also utilized datasets from limited geographical locations, thereby

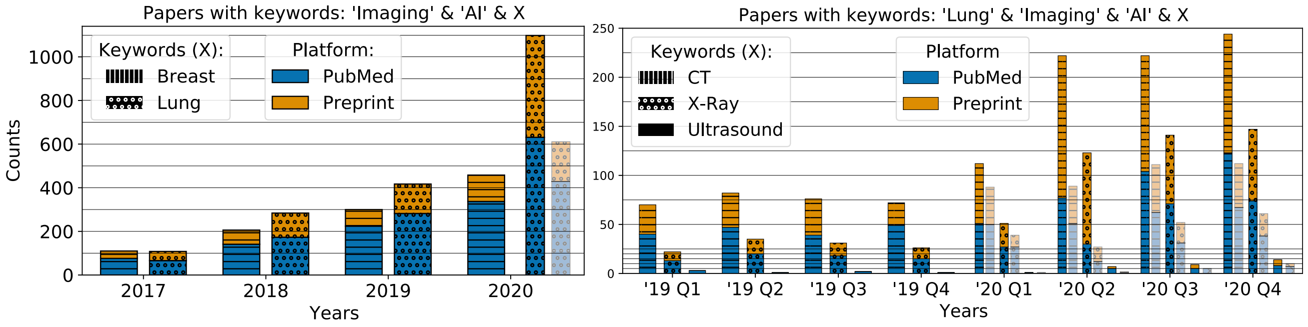

Figure 3. Number of papers per keyword and platform
Left: paper counts using AI on breast or lung imaging. At half-year resolution, the trends persisted; a >100% growth rate for lung was visible in the first half (H1) of 2020 whereas H2 brought about an additional growth of approximately one-third (not shown). The lightly shaded bars exclude COVID-19-related papers, which show the continuity of publications without COVID-19. Right: paper counts comparing the usage of AI on lung imaging modalities. COVID-19 is accompanied by a shift toward more CXR compared with CT papers. For each keyword, multiple synonyms were used (for details see appendix Table A1).

restricting generalization performance of the models in other geographies.

Task-specific review of publications

In this section, we discuss the four categories of tasks addressed by the 463 papers chosen for meta-analysis, namely diagnosis, severity, prognosis, and segmentation. We also highlight key results from the 12 mature publications and provide an overview of the findings specific to COVID-19.

Diagnosis

We find that 72% of the papers centered on COVID-19 diagnosis with 8 out of the 12 mature papers (75%) also addressing this task. As the most prominent COVID-19 test relies on the identification of viral RNA using RT-PCR,[38] imaging is not routinely performed/recommended for diagnosis and, given its reliance on pulmonary pathologies, it is especially inappropriate for detection of early or asymptomatic infections.[39] However, compared with nucleic acid tests, CT may be more sensitive at a single time point for the diagnosis of COVID-19.[40] A key diagnostic challenge is the non-specificity of COVID-19 patterns and their differentiation from non-COVID-19 viral pneumonia.[41] Here, non-imaging assessments such as anamnesis can contribute to the diagnosis. Second, asymptomatic patients with unaffected lungs are notoriously challenging to be detected. In both cases, however, the lack of visibly distinguishing features for COVID-19 might not directly imply a limited ability of DL-based approaches, which might still be able to automatically identify (segment) distinguishing features, given the appropriate data for training.[42]

As has been demonstrated, if DL approaches combine CT and clinical features, the performance of radiologists in the detection of symptomatic COVID-19 patients can be matched[28] (or surpassed[33]), and even asymptomatic patients with normal CT scans can be identified in 68% of the cases.[28]

Moreover, multiple studies validated that radiologists' performance improves upon consultation of AI: Junior radiologists along with AI can perform as well as mid-senior radiologists,[27] and radiologists' sensitivity and specificity can improve by nearly 10% through AI.[43]

In another study, AI recovered full-dose CT from ultra-low-dose CTs with a satisfying acceptance score of 4.4 out of 5 by radiologists (compared with 4.7 and 2.8 for full- and ultra-low-dose, respectively) and thus helped to reduce the CT radiation dose by up to 89% while still facilitating downstream diagnosis.[44] One highly mature diagnostic study using CXR included almost 6,000 scans from >2,000 COVID-19 patients, and their DL model exceeded the diagnostic performance of thoracic radiologists as found by significantly higher AUC of 0.94 (versus 0.85) and sensitivities when matching specificity to radiologists' performance.[35]

Severity assessment

Imaging findings of COVID-19 patients correlate with disease severity,[45] and CT scanning can assess the severity of COVID-19 and help monitor disease transformation among different clinical conditions.[46] A retrospective comparison of imaging findings on chest CTs with disease severity revealed an increased occurrence of consolidation, linear opacities, crazy-paving pattern, and bronchial wall thickening in severe patients at a higher frequency than in non-severe COVID-19 patients. The CT findings correlated with several worse symptoms, including a respiratory rate greater than 30 breaths per minute and oxygen saturation of 93% or less in a resting state, among other phenotypes.[47] In clinical practice, often progress assessments as well as patient management is performed based on CXR and not chest CT. AI that provides assessment of severity could be useful if it was quantifiable and accurate, but only one publication was found to be mature in performing this task.[36] The authors developed a clinically useful AI tool consisting of a UNet backbone for lung segmentation and quantification of pulmonary opacity within 10 days and achieved human-level performance when training on less than 200 CT scans.[36] Another work utilized a dataset of multiple CT scans per patients and introduced a "CT scan simulator" that modeled the temporal evolution of the CT through disease progression and was evaluated on multinational and multimachine data.[48] Their work proposed to decompose the task of CT segmentation from one 3D into three 2D problems, thus achieving remarkable performance. Notably, despite the overall overhead of CXR compared with CT in the analyzed publications, only 3% (n = 6) of the CXR publications in the meta-analysis focused on severity assessment (cf. 14% for CT). One of them trained DL models on lung segmentation and opacity detection of 48 COVID-19 patients and achieved an agreement measure (Cohen's kappa) of 0.51 for alveolar opacities and

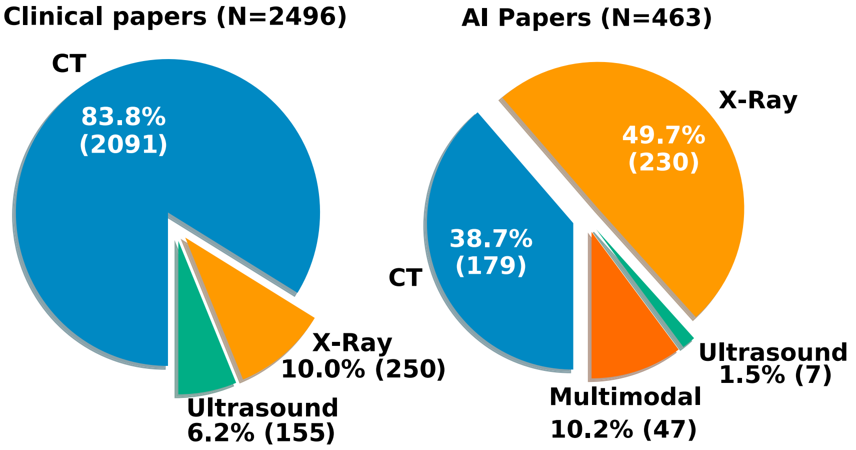

Figure 4. Imaging modality comparison during the COVID-19 pandemic
CT takes the lion's share of clinical papers about lung imaging of COVID-19 (left). The AI community (right) instead published disproportionately more papers on CXR compared with clinicians, whereas CT and ultrasound are under-represented. Multimodal papers used more than one imaging modality.

0.71 for interstitial opacities.[49] In one publication with multimodal imaging data for one patient cohort, manual airspace disease segmentation of CTs in 86 COVID-19 patients was used as ground truth to train a super-resolution convolutional neural network on volumetric quantification from CXR. The obtained correlation percentage of opacity (PO) volume (CT) and PO area (CXR) was around 0.8 for both AI and averaged human experts. A recent study on LUS first inferred a patient-level representation from the region-level LUS videos using attention-based multiple-instance learning and then performed semi-supervised contrastive learning to integrate imaging with clinical data.[50] The method achieved 75% and 88% accuracy in a 4-level/2-level patient severity assessment, respectively, and even identified infected regions in LUS (B-lines) en passant.

Prognosis

Very few of the papers (26, i.e., 6%) focused on prognostic assessments of COVID-19 such as treatment outcome prediction, risk assessment (e.g., requirement for intensive care unit admission or mechanical ventilation), or time elapsed to negative PCR. However, two of them were assessed as mature,[27,29] and the average maturity score was the highest for this task (cf. Figure 6).

However, in contrast to diagnosis, these tasks are clinically more relevant as they cannot be performed routinely and reliably with standard care. While this can be attributed to an overall gap in knowledge of the long-term effects of COVID-19 and a lack of historical data to enable training on large-scale prognosis data, it is constructive toward the alignment of future research in the field. On the other hand, in the past few months the hyper-inflammatory response induced by COVID-19 has been identified as a major cause of disease severity and death.[51] Thus, studies have focused on the identification of predictive biomarkers of pathogenic inflammation. Lung imaging is not expected to reflect these biomarkers' expression, leading to limited prognosis accuracy based on imaging. One study assessed as highly mature seamlessly integrated a diagnostic module (based on a CT lung-lesion segmentation) with a prognostic module that combined clinical metadata and quantification of lung-lesion features.[27] The system demonstrated diagnostic performance comparable with a senior radiologist, and the prognostic module predicted progression to critical illness and could evaluate drug treatment efficacy by three drugs. Notably, the multicenter dataset of 3,777 patients as well as the source code is available to the public to support the development of a better system and to validate their study.

Segmentation

The main abnormalities observed in common and severe COVID-19 cases are ground-glass opacities (GGOs) and patchy consolidation surrounded by GGOs. COVID-19 pneumonia manifests with chest CT imaging abnormalities, even in asymptomatic patients, with rapid evolution from focal unilateral to diffuse bilateral GGOs that progress to or co-exist with consolidations within 1–3 weeks.[52] The visual features of GGOs and consolidation lend themselves to image analysis by DL networks, and with 27 publications (8%) segmentation became the second-most-performed task after diagnosis. In our analysis, many of the papers performed segmentation to enable other clinical tasks as discussed above, but one mature study focused on providing pulmonary lobe segmentation with relational modeling.[30] Using topological modeling techniques that explore structural relationships between vessels, airways, and the pleural wall, and break up with the common strategy of utilizing fully local modules such as convolutions, they achieved human-level performance. In most cases (82%), segmentation publications utilized external data sources with little or no clinical collaboration. Some segmentation-based models output pixelwise-labeled tissue maps of GGO or consolidation regions, providing quantitative localization of findings and identification of disease features, which can be especially informative in clinical tasks such as grading disease severity or tracking progression over time. Chaganti et al. achieved this by segmenting anatomical landmarks with reinforcement learning and computing the PO and lung severity score as complementary severity measures.[53]

In an exhaustive empirical evaluation of DL models on a clinical dataset of almost 100 COVID-19 patients, distinguishing lesion types was found more difficult than lung segmentation or binary lesion segmentation while model ensembles demonstrated best performance.[54] The manual delineation from radiologists, valuable for segmentation tasks, inherently introduces some inter-rater variability, which underlines the need for segmentation techniques that can deal with uncertainty in annotations.[55]

DISCUSSION

In summary, the number of papers on AI in MI for COVID-19 has grown exponentially in 2020, and the quality of the manuscripts varies significantly. In our manual review, only 12 (2.7%) highly mature studies were identified. A key characteristic that underpins

CellPress
OPEN ACCESS

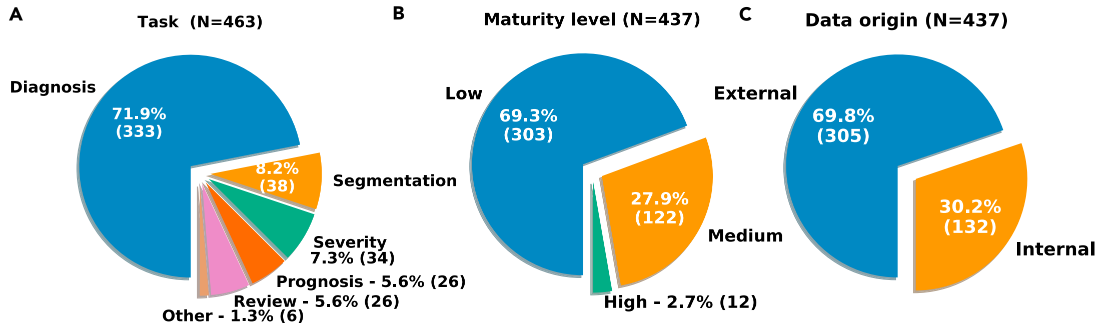

Figure 5. Distribution of manually reviewed papers on AI and MI during the COVID-19 pandemic
Relative proportions for primary performed task (A), quality (B), and data origin (C) are given. N is smaller for (B) and (C), since review papers were excluded from that analysis.

highly mature studies is an interdisciplinary and often multinational collaboration of medical professionals and computer vision researchers.

Challenges and possible solutions

Given the observed disparities between the AI and medical communities, we discuss several challenges that are currently encountered in such interdisciplinary collaborations and provide potential approaches to remedy the same.

Choosing the right task for AI models

The AI literature primarily addresses diagnostic tasks as opposed to other tasks with higher clinical relevance, such as monitoring/severity estimation (which tracks with clinical outcomes) and management tasks such as ventilation equipment and bed allocation. Currently even the best AI solutions have minimal performance gains on well-defined tasks (such as diagnosis) and are thus unlikely to be adopted clinically.[8]

Conclusions from our meta-analysis are that (1) the choice of task is critically driven by the availability of annotated data and (2) the speed of execution in AI propels blind response to increase short-term rewards instead of finding solutions to high-priority problems. This partly explains the overattention to diagnostic tasks. Moreover, classification is the canonical machine-learning (ML) formulation and, while regression techniques can estimate non-binary severity scores, they are less frequently used. Severity estimation can be reduced to summing a classification problem on the pixel level, but this requires very expensive pixelwise annotated training data. Another common misalignment between communities is the disparate objective functions in diagnostic classification of COVID-19 from imaging data. Irrespective of the availability of direct tests for SARS-CoV-2, radiologists around the globe are steered by the objective to avoid false negatives; their decisions are less factious and dichotomous and more granular than a categorical classification of an ML model. On the other hand, the utility of an AI model, trained on a ground truth assigned by radiologists' interpretation, is limited and mostly restricted toward saving time and resources rather than getting better decisions.

To remedy and develop better clinical prediction models, the seven steps for development and four steps for validation proposed by Steyerberg et al.[56] should be followed and complemented by an increased motivation among AI experts to focus on the correct questions and leverage-suitable and radiologist-friendly inductive biases such as soft labeling.[57] Since AI techniques are data driven, the best way to steer AI practice toward more COVID-19 clinical relevance is to collect CT data with annotations for severity as well as demographics data and outcomes data. Recent collaborative, multi-institution data-collection efforts such as the NHS NCCID and RSNA's RICORD datasets precisely have CT data combined with outcomes and severity, and they are sure to lead to AI approaches with more clinical impact. AI challenge competitions are a related route for channeling AI toward CT and severity estimation. MICCAI's COVID-19 lung CT lesion segmentation challenge collected a CT dataset with detailed, radiologist-labeled lesions on the pixel level. AI-based lesion segmentation can then estimate severity by counting lesion voxels. In general, the hope is that this can be applied to longitudinal studies to track COVID-19 progression and eventually be combined with demographics and hospitalization data. Two other promising and clinically relevant endeavors are (1) usage of DL for generating standardized assessment of pulmonary involvement of COVID-19 by leveraging the newly introduced COVID-19 Reporting and Data System (CO-RADS)[34] and (2) using DL to help evaluate treatment outcomes, e.g., by assessing changes in lesion size and volume changes.[27]

Transparency and reproducibility

While most authors of highly mature studies released their code (indeed three papers did not release code[29,31,37]), only one-third of them released at least part of their data. This raises concerns about reproducibility and transparency of their studies, as recently argued against a *Nature* study on breast cancer screening[11] in a "matters arising."[58] Similarly, a COVID-19 mortality prediction study[59] was found to be irreproducible by three independent research groups from different countries.[60–62] Given the global, unprecedented public health challenge caused by COVID-19, we strongly encourage medical researchers to follow the trends toward open-source development in the field of ML (which was proclaimed by various luminaries 14 years ago[63] and successfully implemented in important venues). We encourage researchers to expedite a transformation toward a common practice of validating the proposed methodology and results by publishing both code and, whenever possible, anonymized medical data, especially in academic, non-commercial settings. To help foster this transformation, conference organizers and journal editors should encourage the open sharing of code and anonymized data in their call for papers and add

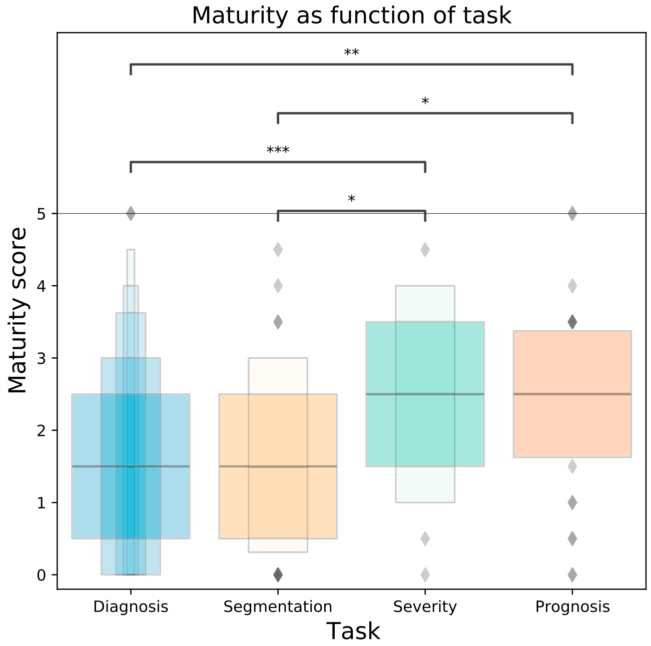

Figure 6. Maturity score as function of task (N = 437)
Publications focusing on COVID-19 diagnosis/detection or pure segmentation achieved a significantly lower maturity score than publications addressing/severity assessment/monitoring or prognostic tasks (asterisks indicate significance levels 0.05, 0.01, and 0.001, respectively).

this as a criterion to the review procedure. For example, NeurIPS and ICML, premier ML conferences, expect that submissions include code and anonymized data and take this into account during the decision-making process. Similarly, the imaging conferences CVPR and MICCAI both strongly encourage the inclusion of code and data. Better guidelines from official sources such as governments are needed, especially since data-sharing regulations are less stringent during a pandemic and medical facilities are often not aware of the numerous advantages of data sharing. Privacy-preserving data science techniques have advanced[64] and should help to build more trust toward data sharing.

Federated learning (FL) is an emerging realm of ML concerned with distributed, decentralized training that stores privacy-sensitive data only locally (for details see Yang et al., Qiang and Zhang, and Vaid et al.[65–67]). FL allows multiple parties to collaboratively train the same model without data sharing and could thus become key to fostering collaborations between clinical and AI communities and overcoming privacy concerns. Our meta-analysis included three preprints exploring FL using CT[68] or CXR[69] data. A recent FL study on electronic health records from five hospitals was found to improve COVID-19 mortality prediction.[70] These efforts will hopefully increase reproducibility and make comparative studies more feasible, which will help the research community focus on the highest-performing methods.

Imaging modality rivalry

An ideal imaging modality should be safe, ubiquitous, accurate, fast, and preferably provide high-quality reproducible results via portable devices. The three different imaging modalities addressed in this study differ in their clinical use, availability, portability, safety, and reproducibility, and none of them is ideal for addressing all aspects of the pandemic (for a comparison see Table 2). For a geographic map showing the regional market sizes of the modalities, see Figure A2. Herein we have unraveled a mismatch in the number of publications per modality between clinical and AI communities: the AI literature has focused mostly on CXR whereas CT and LUS have received comparably little attention (cf. Figure 4). CT is deemed the gold standard, dominates in clinical publications, and is more sensitive than CXR for detecting diseases of the chest, but is restricted to modern medical facilities.[71] CXR is notoriously less sensitive than CT,[72] yet it is the most abundantly used modality across the globe when managing COVID-19 patients. While CXR can underestimate disease, CT can narrow down a differential diagnosis that appears broad on CXR. For AI, large datasets are needed for ML approaches, and there are much larger datasets for CXR than for CT.

As the use of imaging is less regulated compared with PCR/antigen testing, an official recognition of all imaging modalities by leading institutions and stakeholders is needed. In conjunction with clear guidelines for clinicians on when to use which modality, trust in imaging can be increased and workflows can be streamlined. For example, the practical advantages of LUS include non-invasiveness and portability and its consequent role in triage.[73] However, LUS is operator dependent and requires close patient contact for a relatively longer time.[74] It was described as a preferred modality in Italy[75] during spring 2020, but it is not used as extensively in other geographic regions, being mainly applied for patients with CT/CXR contraindications and predestined to study solid organs unlike the lung. Notably, LUS sensitivity was found to be higher than that of CXR for COVID-19 diagnosis,[76] and some even found comparable diagnostic accuracy to CT.[77,78] However, the role of LUS for the COVID-19 pandemic is still actively debated[79–81] and, regarding AI, with only one publicly available dataset,[26] more research is needed to narrow down the practical role of AI on LUS.[26,50,82,83] Additionally, studies using ML on multiple imaging modalities from the same cohort are certainly needed to shed light on comparative questions between modalities from the perspective of ML. The performance of AI-assisted radiologists in detecting COVID-19 might or might not confirm the current radiologic findings, for example that CXR is less sensitive than CT[84] and LUS (when compared with RT-PCR[76] or CT[85]) or that B-lines are the most reliable pathological pattern across CT, CXR, and LUS.[86] From the AI perspective, LUS is presumably the modality with the highest improvement potential in MI analysis in the near future. Ultimately, AI technology focusing on plain CXR/LUS data may enable wider leverage in developing countries with limited medical resources.

ML interpretability

The combined lack of robustness and interpretability poses steep challenges for the adoption of AI models in clinical practice.[87] Models trained without optimizing for reliability typically make overconfident wrong predictions or underconfident correct predictions, especially when extrapolating data. To ensure that models make decisions for the right reasons, they must be trained to recognize out-of-distribution samples and handle distribution shifts, thereby allowing models to abstain from making predictions when it is unsure and deferring such samples to the experts. A human-interpretable access to the model's decision

Table 1. Detailed information on the 12 best papers found in our systematic meta-review of 463 papers (maturity score of high)

Paper title	Primary task; modality	Key findings	Limitations	Patients (train/val/test)	No. of data sites	Labels	Architecture, dimensionality	Pretraining	Metrics	Results	Reproducibility (code/data open source)
Artificial intelligence-enabled rapid diagnosis of patients with COVID-19[27]	diagnosis, CT	system identified 68% of RT-PCR-positive patients with normal CT (asymptomatic). Clinical information is important for diagnosis and model is equally sensitive than a senior radiologist	small data size, mild cases have few abnormal findings on chest CT, severity of pathological findings variable in CT	534/92/279	18	RT-PCR tests	Inception-ResNet-v2 (pretrained ImageNet), 3-layer MLP, 2D	transfer learning (pulmonary tuberculosis model)	AUROC, sensitivity, specificity	0.92 AUC, 84.3% sens, 82.8% spec	code—yes, data—no
Artificial intelligence augmentation of radiologist performance in distinguishing COVID-19 from pneumonia of other origin at chest CT[32]	Diagnosis, CT	AI assistance improved radiologists' performance in diagnosing COVID-19. AI alone outperformed radiologists on sensitivity and specificity	bias in radiologist-annotation, heterogeneous data, bias in location of COVID (China) versus non-COVID pneumonia patients (USA)	830/237/119	13	RT-PCR tests, slice-level by radiologist	EfficientNet-B4, 2D	transfer learning (ImageNet)	AUROC, sensitivity, specificity, accuracy, AUPRC	0.95 AUC, 95% sens, 96% spec, 96% acc, 0.9 AUPRC	code—yes, data—no
Automated assessment of CO-RADS and chest CT severity scores in patients with suspected COVID-19 using artificial intelligence[33]	diagnosis, CT	a freely accessible algorithm that assigns CO-RADS and CT severity scores to non-contrast CT scans of patients suspected of COVID-19 with high diagnostic performance	only one data center, high COVID prevalence, low prevalence for other diseases	476/105	1	RT-PCR, radiology report	lobe segmentation 3D UNet, CO-RADS scoring, 3D Inception Net	transfer learning (ImageNet and kinetics)	AUC, sensitivity, specificity	internal: 0.95 AUC, external: 0.88 AUC	code—yes, data—no

(*Continued on next page*)

Table 1. *Continued*

Paper title	Primary task; modality	Key findings	Limitations	Patients (train/val/test)	No. of data sites	Labels	Architecture, dimensionality	Pretraining	Metrics	Results	Reproducibility (code/data open source)
Diagnosis of Covid-19 pneumonia using chest radiography: value of artificial intelligence[35]	diagnosis, X-ray	AI surpassed senior radiologists in COVID-19 differential diagnosis	high COVID prevalence, human ROC-AUC were averaged from 3 readers	5,208/ 2,193	5 hospitals, 30 clinics	RT-PCR, natural language processing on radiology report	CV19-Net	3-stage transfer learning (ImageNet)	AUC, sensitivity, specificity	0.92 AUC, 88.0% sens, 79.0% spec	code—yes, data—no
Development and evaluation of an artificial intelligence system for COVID-19 diagnosis[23]	diagnosis, multimodal	paired cohort of chest X-ray (CXR)/CT data: CT is superior to CXR for diagnosis by wide margin. AI system outperforms all radiologists in 4-class classification	more data on more pneumonia subtypes needed, no clinical information used (could enable severity assessment)	2,688/ 2,688/ 3,649	7	–	lung seg 2D UNet, slice diagnosis 2D ResNet152	transfer learning (pretrained ImageNet)	AUC, sensitivity, specificity	AUC 0.978	code—yes, data—no
AI-assisted CT imaging analysis for COVID-19 screening: building and deploying a medical AI system[31]	diagnosis, CT	system was deployed in 4 weeks in 16 hospitals; AI outperformed radiologists in sensitivity by wide margin	model fails when multiple lesions, metal or motion artifacts are present, system depends on fully annotated CT data	1,136	5	Nucleic acid test, 6 annotators (lesions, lung)	3D UNet++, ResNet50	full training	sensitivity, specificity	sens 97.4%, spec 92.2%	code—no, data—no

(*Continued on next page*)

Table 1. *Continued*

Paper title	Primary task; modality	Key findings	Limitations	Patients (train/ val/test)	No. of data sites	Labels	Architecture, dimensionality	Pretraining	Metrics	Results	Reproducibility (code/data open source)
Automated assessment and tracking of COVID-19 pulmonary disease severity on chest radiographs using convolutional Siamese neural networks[32]	severity, X-ray	continuous severity score used for longitudinal evaluation and risk stratification (admission CXR score predicts intubation and death, AUC = 0.8). Follow-up CXR score by AI is concordant with radiologist (r = 0.74)	patients only from urban areas in USA, no generalization to posteroanterior radiographs	160,000/ 267 (images)	2	RT-PCR tests, 2–5 annotators, mRALE	Siamese DenseNet-121	DenseNet-121 (ImageNet, fine-tuned on CheXpert)	PXS score, Pearson, AUC	r = 0.86, AUC = 0.8	code—yes, data—partial (COVID CXR not released)
Development and clinical implementation of tailored image analysis tools for COVID-19 in the midst of the pandemic[36]	severity, CT	developed algorithms for quantification of pulmonary opacity in 10 days. Human-level performance with <200 CT scans. Model integrated into clinical workflow	data: no careful acquisition, not complete, consecutively acquired or fully random sample; empirical HU-thresholds for quantification	146/66	1	RT-PCR, 3 radiologist annotators	3D UNet	full training	Dice coefficient, Hausdoff distance	Dice = 0.97	code—yes, data—no

(*Continued on next page*)

CellPress
OPEN ACCESS

Table 1. *Continued*

Paper title	Primary task; modality	Key findings	Limitations	Patients (train/val/test)	No. of data sites	Labels	Architecture, dimensionality	Pretraining	Metrics	Results	Reproducibility (code/data open source)
Clinically applicable AI system for accurate diagnosis, quantitative measurements, and prognosis of COVID-19 pneumonia using computed tomography[27]	prognosis, CT	AI with diagnostic performance comparable with senior radiologist. AI lifts junior radiologists to senior level. AI predicts drug efficacy and clinical prognosis. Identifies biomarkers for novel coronavirus pneumonia lesion. Data available		3,777	4	pixel-level annotation (5 radiologists)	lung-lesion seg DeepLabV3, diagnosis analysis 3D ResNet-18, gradient boosting decision tree	full training	Dice coefficient, AUC, accuracy, sensitivity, specificity	AUC 0.9797, acc 92.49%, sens 94.93%, spec 91.13%	code—yes, data—yes
Relational modeling for robust and efficient pulmonary lobe segmentation in CT scans[30]	segmentation, CT	leverages structured relationships with non-local module. Can enlarge receptive field of convolution features. Robustly segments COVID-19 infections	errors on border of segmentations, gross pathological changes not represented in data	4,370/ 1,100	2 (pretraining: 21 centers)	radiology report	RTSU-Net (2-stage 3D UNet)	pretraining on COPDGene	intersection over union, average asymmetric surface distance	IOU 0.953, AASD 0.541	code—yes, data—no/partial

(*Continued on next page*)

Table 1. *Continued*

Paper title	Primary task; modality	Key findings	Limitations	Patients (train/val/test)	No. of data sites	Labels	Architecture, dimensionality	Pretraining	Metrics	Results	Reproducibility (code/data open source)
Dual-branch combination network (DCN): toward accurate diagnosis and lesion segmentation of COVID-19 using CT images[37]	diagnosis, CT	DCN for combined segmentation and classification. Lesion attention (LA) module improves sensitivity to CT images with small lesions and facilitates early screening. Interpretability: LA provides meaningful attention maps	diagnosis depends on accuracy of segmentation module, no slice-level annotation	1,202	10	RT-PCR, pixel-level annotation by 6 radiologists	UNet, ResNet-50	full training	accuracy, Dice, sensitivity, specificity, AUC, average accuracy	acc 92.87%, Dice 99.11%, sens 92.86%, spec 92.91%, AUC 0.977, average acc 92.89%	code—no, data—no
AI-driven quantification, staging and outcome prediction of COVID-19 pneumonia[29]	prognosis, CT	2D/3D COVID-19 quantification, roughly on par with radiologists. Facilitates prognosis/staging which outperforms radiologists. Rich set of model ensembles, uses clinical features	test dataset partly split by centers	693 (321,000 slices)/513 for test	8	RT-PCR	AtlasNet, 2D	full training	Dice coefficient, correlation, accuracy	Dice 0.7, balanced accuracy 0.7	code—no, data—yes (without images)

For discussion, please see the text.

CellPress
OPEN ACCESS

Table 2. Differences between the imaging modalities

	CT	CXR	LUS
Benefit	• high sensitivity<br>• high specificity	• fast<br>• broadly available	• portable<br>• radiation-free<br>• broadly available
Drawback	• patient transportation<br>• low availability<br>• radiation dose<br>• increased workload for disinfection	• low sensitivity<br>• non-specific<br>• large volume of radiographs leads to increased workload	• user-dependent<br>• non-specific<br>• long acquisition time<br>• requires patient interaction
Clinical role	• diagnose additional complications<br>• rule out additional etiologies of symptoms (effusions, bacterial pneumonia)	• initial diagnosis<br>• monitoring clinical progression<br>• detection of complications	• triage<br>• point-of-care monitoring for specific tasks

process is crucial to hone trust in AI, especially in medical applications where reasoning is inductive, sensitive decisions are made, and patients expect plausible hypotheses from physicians. In MI, heatmap techniques (such as GradCAM[88] or guided-backpropagation[89]) and uncertainty estimation of individual predictions (e.g., with MC Dropout[90,91] or test-time-augmentation[92]) are the most widely adopted approaches. However, most current interpretability tools focus on generating explanations that highlight patterns learned from the data but do not translate model decisions in human-understandable forms. Counterfactual reasoning has found its way into ML explainability[93] and has opened doors toward contrastive explanations (by ascribing how changing the input would affect predictions), and can readily be combined with uncertainty quantification principles to build models integrating reliability into the optimization process.[94] This will enable model introspection and facilitate human-in-the-loop analysis while also considering the performance distribution among human evaluators.

Collaboration between AI and clinical communities

A standard healthcare AI project workflow involves defining a use-case, curating data and annotations, identifying problem constraints, choosing relevant metrics, designing and building the AI system, and lastly evaluating the model performance (Figure 7, top). However, any problem involves many stakeholders: patients, ethics committees, regulatory bodies, hospital administrators, clinicians, and AI experts.[95] In general, data-driven constraints identified by the AI experts tend to transform the clinical task into an evolved task. In combination with the disconnect of other parties (e.g., clinicians, patients) in the build life cycle, this causes potential gaps in the overall outcomes of the collaboration. Awareness and understanding of the difference in needs, motivations, and solution interpretations across agents is imperative. For example, for clinicians the generation of data and metadata are cumbersome, time demanding, and tedious. What drives and motivates clinicians are improved clinical workflows and the knowledge and better understanding the analysis can bring, so that they can provide improved patient care. Moreover, AI models may hide inherent risks such as the codification of biases, the weak accountability, and the bare transparency of their decision-making process. Therefore, the way AI models are evaluated can have multiple implications on their applicability, generalization, and translation to clinical practice.[96,96] To this end, both the definition of the task to be implemented and evaluated, but also the types of metrics to be leveraged to evaluate the results' outcomes, can be different across collaborators and hence must be collectively defined.

We illustrate such an improved workflow that incorporates other stakeholders in the build process, robust metrics, and iterative usability studies in Figure 7 (bottom). We believe that such a workflow could critically improve the quality of collaboration between AI and clinicians.

To enable agile and transparent development with continuous feedback and evaluation loops, new conducive environments are necessary. A collaboration environment that enables sharing of data, code, and results, but also immediate feedback and discussion platforms across collaborators, is essential. Communities of discovery such as the digital mammography DREAM challenge[97] that bring together experts across domains under a unified cloud-based platform can enable data privacy and compliance through distributed learning and FL. Data and code sharing through open-source and open-access initiatives, and comprehensive, multidisciplinary validation could pave the way toward closing the gap between technology development and translation to clinical practice.

To summarize, the challenges toward improved collaboration include (1) aligning goals of diverse stakeholders (e.g., clinicians, AI experts, patients, funding and regulatory agencies) and (2) mapping a medical need into a well-defined task with a measurable and applicable outcome. Possible solutions include (1) inclusive execution and transparency (e.g., keep clinicians and/or patients involved throughout the build process), (2) robust evaluation of systems (e.g., going beyond accuracy metrics to incorporate reliability metrics), and (3) creation of common work environments.

Despite the scientometric research which revealed that during COVID-19 global research investments and publication efforts have grown dramatically,[98] research team sizes, number of involved countries, and ratio of international collaborations shrank.[99] We therefore hope to encourage more international collaborations between the AI community and medical experts, as this could lead to more mature and conducive technologies and potentially assist clinicians and radiologists in addressing pressing clinical decision support needs during the pandemic.

EXPERIMENTAL PROCEDURES

Resource availability

Lead contact

Jannis Born (jab@zurich.ibm.com).

Materials availability

Not applicable.

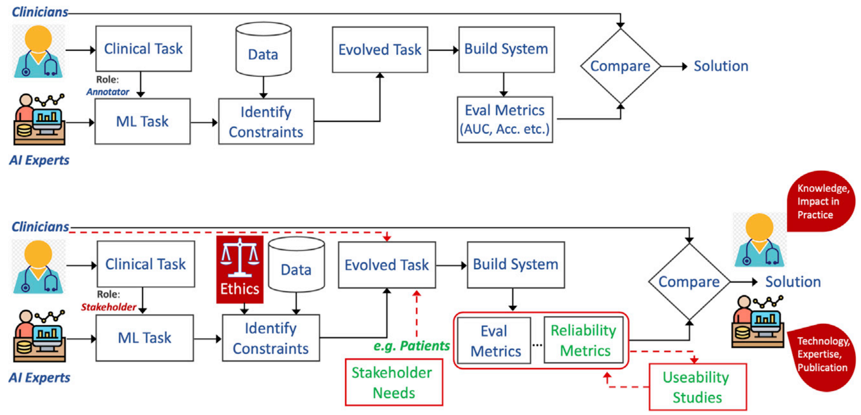

Figure 7. Workflow of collaboration between AI and clinical experts
Top: typical process of developing healthcare AI technology including task definition, data curation, building ML systems, and human-in-the-loop evaluation. Bottom: our proposed workflow, highlighting key components that need to be incorporated into the process to improve collaboration between AI and clinical experts. Note the disparity in value interpretation of the developed solutions by the two communities.

Data and code availability

The source code used for the publication keyword search is available via https://pypi.org/project/paperscraper/. A spreadsheet with the detailed results of the publication meta-analysis is enclosed as supplemental information (online only).

Methods

To discover trends from the overwhelming research activities in COVID-19, AI and MI, we performed a systematic review and meta-analysis according to the PRISMA guidelines.[100] Literature, indexed in PubMed and three preprint servers, namely arXiv, bioRxiv, and medRxiv, were queried. The process is illustrated in Figure 1 (left) and shows two main streams of queries: a broad one using "AI" AND "COVID-19" AND "Medical Imaging" and a modality-specific one with "AI" AND "COVID-19" AND "Lung" AND ("CT" OR "CXR" OR "US"). Following PRISMA guidelines, we combined the results of both queries across all databases leading to the identification of 463 papers about AI on lung imaging for COVID-19. These papers were included in a manual meta-analysis to review the maturity of the AI technologies and the trends in the rapidly evolving field (for the detailed procedure and a list of synonyms used, see appendix Table A1). The publications about AI technology typically tend to report a proof of concept, an illustration of a success in a non-clinical setting, or a report of clinically successful experiments. Additionally, many of the papers identified were not published in peer-reviewed journals. To evaluate the maturity of papers, we included five criteria that were assessed rigorously (Figure 1, right).

1. Peer review: Whether or not the paper appeared in a peer-reviewed journal or conference.
2. Modeling quality: The complexity and the performance of the developed AI framework.
3. Data quality/scale: Number of patients in the data used for training and evaluation. Internal, clinical data is preferred over public datasets, and multihospital/multimodal data are valued.
4. Experimental rigor: Stringency in the evaluation and comparison of the methodology.
5. Clinical deployment: The deployment and adoption of the solution in hospitals. Comparison studies of AI and radiologists or deployment of web services were also rewarded.

The peer-review score was binary and all other categories were scored ternarily (0, 0.5, 1). Details of the scheme with examples can be found in the supplemental information. The decision function for maturity level (Figure 1, right) guarantees that publications which received a "0" in one of the five categories cannot obtain a high maturity score (implying that, e.g., preprints are never highly mature).

Moreover, we manually inferred the most common tasks addressed in the AI papers, such as detection, segmentation, characterization, and outcome prediction, and mapped them into three main clinically relevant categories—diagnosis, severity assessment, and prognosis—and one technical task, segmentation. The segmentation papers discuss localization of lung tissue or other disease features without direct applications to any clinically relevant downstream tasks.

For publications that focused on several categories, we consider the primary task only. For example, a number of publications classified as "diagnosis" or "severity assessment" utilized segmentation methods on the fly. Papers that provided a review of ML for MI on COVID-19 and did not introduce original new technology were labeled as "review" papers and excluded from the maturity assessment, leading to 437 reviewed papers. The remaining evaluation criteria per publication were imaging modality, country of authors, and country of data source. For each paper, we also recorded the total number of citations indicated on Google Scholar as of February 28, .2021 and converted it to the monthly citation rate. Note that the meta-analysis was blindfolded to the number of citations.

The publication keyword search was performed using our toolbox *paperscraper* that was developed during this project and is open-sourced (https://pypi.org/project/paperscraper/).

SUPPLEMENTAL INFORMATION

Supplemental information can be found online at https://doi.org/10.1016/j.patter.2021.100269.

AUTHOR CONTRIBUTIONS

Conceptualization, M.R.-Z., D. Beymer, J.L.R., E.K., M. Gabrani, J.B., and D.R.; Methodology, M.R.-Z., M. Gabrani, D. Beymer, J.B., and D.R.; Software, J.B. and M.M.; Validation, M.R.-Z., M. Gabrani, D. Beymer, and J.B.; Formal analysis, J.B. and D.R.; Investigation, J.B., D.R., D. Beymer, A.C., V.V.M., E.K., and M. Gabrani; Data curation, J.B.; Writing – original draft, J.B., D. Beymer, D.R., M. Gabrani, M.R.-Z., J.L.R., E.K., A.C., P.L.S., D.S., and M. Guindy; Writing – review & editing, J.B., D.R., M. Gabrani, D. Beymer, and M.R.-Z.; Visualization, J.B., D.R., and M. Gabrani; Supervision, M.R.-Z., M. Gabrani, J.L.R., E.K., P.L.S., A.C., D.S., M. Guindy, V.V.M., P.P., and D. Ballah; Project administration, M.R.-Z., M. Gabrani, and D. Beymer.

DECLARATION OF INTERESTS

The authors declare no competing interests.

REFERENCES

1. Dong, D., et al. (2020). The role of imaging in the detection and management of COVID-19: a review. IEEE Rev. Biomed. Eng. *14*, 16–29. https://doi.org/10.1109/RBME.2020.2990959.

2. Pascarella, G., et al. (2020). "COVID-19 diagnosis and management: a comprehensive review. J. Intern. Med. 288, 192–206.

3. Rubin, G.D., Ryerson, C.J., Haramati, L.B., Sverzellati, N., Kanne, J.P., Raoof, S., et al. (2020). The role of chest imaging in patient management during the COVID-19 pandemic: a multinational consensus statement from the Fleischner Society. Chest *158*, 106–116. https://doi.org/10.1016/j.chest.2020.04.003.

4. American College of Radiology (2020). ACR recommendations for the use of chest radiography and computed tomography (CT) for suspected COVID-19 infection, Updated March, 22 https://www.acr.org/Advocacy-and-Economics/ACR-Position-Statements/Recommendations-for-Chest-Radiography-and-CT-for-Suspected-COVID19-Infection.

5. Lomoro, P., et al. (2020). COVID-19 pneumonia manifestations at the admission on chest ultrasound, radiographs, and CT: single-center study and comprehensive radiologic literature review. Eur. J. Radiol. Open *7*, 100231. https://doi.org/10.1016/j.ejro.2020.100231.

6. Shi, F., Wang, J., Shi, J., Wu, Z., Wang, Q., Tang, Z., et al. (2020). Review of artificial intelligence techniques in imaging data acquisition, segmentation and diagnosis for Covid-19. IEEE Rev. Biomed. Eng. *14*, 4–15. https://doi.org/10.1109/RBME.2020.2987975.

7. L. Wynants *et al*., "Prediction models for diagnosis and prognosis of Covid-19: systematic review and critical appraisal," BMJ, vol. 369, p. 18, Apr. 20 .

8. Celi, L.A., Fine, B., and Stone, D.J. (2019). An awakening in medicine: the partnership of humanity and intelligent machines. Lancet Digit. Health *1*, e255–e257.

9. Kim, H.E., et al. (2020). Changes in cancer detection and false-positive recall in mammography using artificial intelligence: a retrospective, multireader study. Lancet Digit. Heal. *2*, e138–e148.

10. Akselrod-Ballin, A., et al. (2019). Predicting breast cancer by applying deep learning to linked health records and mammograms. Radiology *292*, 331–342.

11. McKinney, S.M., et al. (2020). International evaluation of an AI system for breast cancer screening. Nature *577*, 89–94.

12. Le, E.P.V., Wang, Y., Huang, Y., Hickman, S., and Gilbert, F.J. (2019). Artificial intelligence in breast imaging. . Clinical Radiology, *74* (W.B. Saunders Ltd), pp. 357–366.

13. Halling-Brown, M.D., et al. (2020). OPTIMAM mammography image database: a large-scale resource of mammography images and clinical data. Radiol. Artif. Intell. e200103.

14. Irvin, J., et al. (2019). CheXpert: a large chest radiograph dataset with uncertainty labels and expert comparison. Proc. AAAI Conf. Artif. Intell. *33*, 590–597.

15. Ardila, D., et al. (2019). End-to-end lung cancer screening with three-dimensional deep learning on low-dose chest computed tomography. Nat. Med. *25*, 954–961.

16. Saba, L., Biswas, M., Kuppili, V., Godia, E.C., Suri, H.S., Edla, D.R., Omerzu, T., Laird, J.R., Khanna, N.N., Mavrogeni, S., and Protogerou, A. (2019). The present and future of deep learning in radiology. Eur. J. Radiol. *114*, 14–24.

17. Syeda, H.B., et al. (2021). Role of machine learning techniques to tackle the COVID-19 crisis: systematic review. JMIR Med. Inform. *9*, e23811.

18. Chiroma, H., et al. (2020). Early survey with bibliometric analysis on machine learning approaches in controlling COVID-19 outbreaks. PeerJ Computer Sci. *6*, e313.

19. Albahri, O.S., et al. (2020). Systematic review of artificial intelligence techniques in the detection and classification of COVID-19 medical images in terms of evaluation and benchmarking: Taxonomy analysis, challenges, future solutions and methodological aspects. J. Infect. Public Health *13*, 1381–1396. https://doi.org/10.1016/j.jiph.2020.06.028.

20. Roberts, M., et al. (2021). Common pitfalls and recommendations for using machine learning to detect and prognosticate for COVID-19 using chest radiographs and CT scans. Nat. Machine Intelligence *3*, 199–217.

21. Gupta, A., et al. (2020). Extrapulmonary manifestations of COVID-19. Nat. Med. *26*, 1017–1032.

22. Barbosa, E.J.M., Jr., et al. (2021). Automated detection and quantification of COVID-19 airspace disease on chest radiographs: a novel approach achieving expert radiologist-level performance using a deep convolutional neural network trained on digital reconstructed radiographs from computed tomography-derived ground truth. Invest. Radiol., In press. https://doi.org/10.1097/RLI.0000000000000763.

23. Jin, C., et al. (2020). Development and evaluation of an artificial intelligence system for COVID-19 diagnosis. Nat. Commun. *11*, 5088.

24. Soares, E., et al. (2020). SARS-CoV-2 CT-scan dataset: a large dataset of real patients CT scans for SARS-CoV-2 identification. medRxiv. https://doi.org/10.1101/2020.04.24.20078584.

25. Cohen, J.P., et al. (2020). Covid-19 image data collection: prospective predictions are the future. Journal of Machine Learning for Biomedical Imaging (MELBA), arXiv:2006.11988.

26. Born, J., Wiedemann, N., Cossio, M., Buhre, C., Brändle, G., Leidermann, K., Aujayeb, A., Moor, M., Rieck, B., and Borgwardt, K. (2021). Accelerating detection of lung pathologies with explainable ultrasound image analysis. Appl. Sci. *11*, 672. https://doi.org/10.3390/app11020672.

27. Zhang, K., Liu, X., Shen, J., Li, Z., Sang, Y., Wu, X., et al. (2020). Clinically applicable AI system for accurate diagnosis, quantitative measurements, and prognosis of COVID-19 pneumonia using computed tomography. Cell *181*, 1360.

28. Mei, X., Lee, H.-C., Diao, K.-Y., Huang, M., Lin, B., Liu, C., et al. (2020). Artificial intelligence-enabled rapid diagnosis of patients with COVID-19. Nat. Med. *26*, 1224–1228.

29. Chassagnon, G., et al. (2020). AI-driven quantification, staging and outcome prediction of COVID-19 pneumonia. Med. Image Anal. *67*, 101860.

30. Xie, W., et al. (2020). Relational modeling for robust and efficient pulmonary lobe segmentation in CT scans. IEEE Trans. Med. Imaging *39*, 2664–2675.

31. Wang, B., et al. (2020). AI-assisted CT imaging analysis for COVID-19 screening: building and deploying a medical AI system. Appl. Soft Comput. *98*, 106897.

32. Li, M.D., et al. (2020). Automated assessment and tracking of COVID-19 pulmonary disease severity on chest radiographs using convolutional Siamese neural networks. Radiol. Artif. Intelligence *2*, e200079.

33. Bai, H.X., Wang, R., Xiong, Z., Hsieh, B., Chang, K., Halsey, K., et al. (2020). Artificial intelligence augmentation of radiologist performance in distinguishing COVID-19 from pneumonia of other origin at chest CT. Radiology *296*. https://doi.org/10.1148/radiol.2021219004.

34. Lessmann, N., Sánchez, C.I., Beenen, L., Boulogne, L.H., Brink, M., Calli, E., et al. (2020). Automated assessment of CO-RADS and chest CT severity scores in patients with suspected COVID-19 using artificial intelligence. Radiology, 202439.

35. Zhang, R., et al. (2020). Diagnosis of covid-19 pneumonia using chest radiography: value of artificial intelligence. Radiology, 202944.

36. Anastasopoulos, C., Weikert, T., Yang, S., Abdulkadir, A., Schmülling, L., Bühler, C., et al. (2020). Development and clinical implementation of tailored image analysis tools for COVID-19 in the midst of the pandemic: the synergetic effect of an open, clinically embedded software development platform and machine learning. Eur. J. Radiol. *131*, 109233.

37. Gao, K., et al. (2020). Dual-branch combination network (DCN): towards accurate diagnosis and lesion segmentation of COVID-19 using CT images. Med. image Anal. *67*, 101836.

38. Pascarella, G., et al. (2020). COVID-19 diagnosis and management: a comprehensive review. J. Intern. Med. *288*, 192–206.

39. Tang, Y.W., Schmitz, J.E., Persing, D.H., and Stratton, C.W. (2020). Laboratory diagnosis of COVID-19: current issues and challenges. J. Clin. Microbiol. *58*. https://doi.org/10.1128/JCM.00512-20.

40. Ai, T., et al. (2020). Correlation of chest CT and RT-PCR testing in coronavirus disease 2019 (COVID-19) in China: a report of 1014 cases. Radiology, 200642.

41. Mendel, J.B., Lee, J.T., and Rosman, D. (2020). Current concepts imaging in COVID-19 and the challenges for low and middle income countries. J. Glob. Radiol.

42. Li, L., et al. (2020). Artificial intelligence distinguishes Covid-19 from community acquired pneumonia on chest CT. Radiology *296*, E65–E72. https://doi.org/10.1148/radiol.2020200905.

43. Song, J., et al. (2020). End-to-end automatic differentiation of the coronavirus disease 2019 (COVID-19) from viral pneumonia based on chest CT. Eur. J. Nucl. Med. Mol. Imaging.

44. Shiri, I., et al. (2020). Ultra-low-dose chest CT imaging of COVID-19 patients using a deep residual neural network. Eur. Radiol. *31*, 1420–1431. https://doi.org/10.1007/s00330-020-07225-6.

45. World Health Organization, Use of chest imaging in COVID-19: a rapid advice guide, 2020.

46. Tabatabaei, S.M.H., Talari, H., Moghaddas, F., and Rajebi, H. (2020). Computed tomographic features and short-term prognosis of coronavirus disease 2019 (COVID-19) pneumonia: a single-center study from Kashan, Iran. Radiol. Cardiothorac. Imaging *2*, e200130.

47. Li, M., et al. (2020). Coronavirus disease (COVID-19): spectrum of CT findings and temporal progression of the disease. Acad. Radiol. *27*, 603–608.

48. Zhou, L., et al. (2020). A rapid, accurate and machine-agnostic segmentation and quantification method for CT-based COVID-19 diagnosis. IEEE Trans. Med. Imaging *39*, 2638–2652.

49. Blain, M., et al. (2021). Determination of disease severity in COVID-19 patients using deep learning in chest X-ray images. Diagn. Interv. Radiol. *27*, 20.

50. Xue, W., Cao, C., Liu, J., Duan, Y., Cao, H., Wang, J.,., and Xie, M. (2021). Modality alignment contrastive learning for severity assessment of COVID-19 from lung ultrasound and clinical information. Med. Image Anal. *69*, 101975.

51. Del Valle, D.M., Kim-Schulze, S., Huang, H.H., Beckmann, N.D., Nirenberg, S., Wang, B., Lavin, Y., Swartz, T.H., Madduri, D., Stock, A., and Marron, T.U. (2020). An inflammatory cytokine signature predicts COVID-19 severity and survival. Nat. Med. *26*, 1636–1643.

52. Shi, H., et al. (2020). Radiological findings from 81 patients with COVID-19 pneumonia in Wuhan, China: a descriptive study. Lancet Infect. Dis. *20*, 425–434.

53. Chaganti, S., et al. (2020). Automated quantification of CT patterns associated with COVID-19 from chest CT. Radiol. Artif. Intelligence *2*, e200048.

54. Tilborghs, S., et al. (2020). Comparative study of deep learning methods for the automatic segmentation of lung, lesion and lesion type in CT scans of COVID-19 patients. arXiv, arXiv:2007.15546.

55. Wang, G., et al. (2020). A noise-robust framework for automatic segmentation of covid-19 pneumonia lesions from ct images. IEEE Trans. Med. Imaging *39*, 2653–2663.

56. Steyerberg, E.W., and Vergouwe., Y. (2014). Towards better clinical prediction models: seven steps for development and an ABCD for validation. Eur. Heart J. *35*, 1925–1931.

57. Nguyen, Q., Valizadegan, H., and Hauskrecht, M. (2014). Learning classification models with soft-label information. J. Am. Med. Inform. Assoc. *21*, 501–508.

58. Haibe-Kains, B., et al. (2020). Transparency and reproducibility in artificial intelligence. Nature *586*, E14–E16.

59. Yan, Li, et al. (2020). An interpretable mortality prediction model for COVID-19 patients. Nat. Mach. Intell. *2*, 283–288.

60. Barish, M., et al. (2021). External validation demonstrates limited clinical utility of the interpretable mortality prediction model for patients with COVID-19. Nat. Mach. Intell. *3*, 25–27.

61. Quanjel, M.J.R., et al. (2020). Replication of a mortality prediction model in Dutch patients with COVID-19. Nat. Mach. Intell. *2*, 23–24.

62. Dupuis, C., et al. (2020). Limited applicability of a COVID-19 specific mortality prediction rule to the intensive care setting. Nat. Mach. Intell. *2*, 20–22.

63. Sonnenburg, S., et al. (2007). The need for open source software in machine learning. J. Mach. Learn. Res. *8*, 2443–2466.

64. Liang, X., et al. (2017). Integrating blockchain for data sharing and collaboration in mobile healthcare applications. In IEEE 28th Annual International Symposium on Personal, Indoor, and Mobile Radio Communications (PIMRC) (IEEE). https://doi.org/10.1109/PIMRC.2017.8292361.

65. Yang, Q., et al. (2019). Federated machine learning: concept and applications. ACM Trans. Intell. Syst. Technology (Tist) *10*, 1–19.

66. Qian, F., and Zhang, A. (2021). The value of federated learning during and post COVID-19. Int. J. Qual. Health Care *33*, 1353–4505. https://doi.org/10.1093/intqhc/mzab010.

67. Vaid, A., Jaladanki, S.K., Xu, J., Teng, S., Kumar, A., Lee, S.,., and Glicksberg, B.S. (2021). Federated learning of electronic health records to improve mortality prediction in hospitalized patients with COVID-19: machine learning approach. JMIR Med. Inform. *9*, e24207.

68. Kumar, R., et al. (2020). Blockchain-federated-learning and deep learning models for covid-19 detection using ct imaging. arXiv, arXiv:2007.06537.

69. Liu, B., et al. (2020). Experiments of federated learning for covid-19 chest x-ray images. arXiv, arXiv:2007.05592.

70. Vaid, A., Jaladanki, S.K., Xu, J., Teng, S., Kumar, A., Lee, S.,., and Glicksberg, B.S. (2021). Federated learning of electronic health records to improve mortality prediction in hospitalized patients with COVID-19: machine learning approach. JMIR Med. Inform. *9*, e24207.

71. Castillo, M. (2012). The industry of CT scanning. Am. Soc. Neuroradiol. *33*, 583–585. https://doi.org/10.3174/ajnr.A2742.

72. Wong, H.Y.F., et al. (2019). Frequency and distribution of chest radiographic findings in COVID-19 positive patients. Radiology, 201160.

73. Smith, M.J., Hayward, S.A., Innes, S.M., and Miller, A. (2020). Point-of-care lung ultrasound in patients with COVID-19—a narrative review. Anaesthesia *75*, 1096–1104.

74. Akl, E.A., Blazic, I., Yaacoub, S., Frija, G., Chou, R., Appiah, J.A., Fatehi, M., Flor, N., Hitti, E., Jafri, H., et al. (2020). Use of chest imaging in the diagnosis and management of COVID-19: a WHO rapid advice guide. Radiology, 203173. https://doi.org/10.1148/radiol.2020203173.

75. Vetrugno, L., et al. (2020). Our Italian experience using lung ultrasound for identification, grading and serial follow-up of severity of lung involvement for management of patients with COVID-19. Echocardiography *37*, 625–627.

76. Pare, J.R., Camelo, I., Mayo, K.C., Leo, M.M., Dugas, J.N., Nelson, K.P., et al. (2020). Point-of-care lung ultrasound is more sensitive than chest radiograph for evaluation of COVID-19. West. J. Emerg. Med. Integr. Emerg. Care Popul. Heal. *21*, 771–778. https://doi.org/10.5811/westjem.2020.5.47743.

77. Lieveld, A.W.E., et al. (2020). Diagnosing COVID-19 pneumonia in a pandemic setting: lung Ultrasound versus CT (LUVCT) A multi-centre, prospective, observational study. ERJ Open Res. *6*, 00539–02020. https://doi.org/10.1183/23120541.00539-2020.

78. Tung-Chen, Y., Martí de Gracia, M., Díez-Tascón, A., Alonso-González, R., Agudo-Fernández, S., Parra-Gordo, M.L., Ossaba-Vélez, S., Rodríguez-Fuertes, P., and Llamas-Fuentes, R. (2020). Correlation between chest computed tomography and lung ultrasonography in patients with coronavirus disease 2019 (COVID-19). Ultrasound Med. Biol. *46*, 2918–2926.

79. Tung-Chen, Y., et al. (2020). Correlation between chest computed tomography and lung ultrasonography in patients with coronavirus disease 2019 (COVID-19). Ultrasound Med. Biol. *46*, 2918–2926. https://doi.org/10.1016/j.ultrasmedbio.2020.07.003.

80. Buonsenso, D., Pata, D., and Chiaretti, A. (2020). COVID-19 outbreak: less stethoscope, more ultrasound. Lancet Respir. Med. *8*. https://doi.org/10.1016/S2213-2600(20)30120-X.

81. Cheung, J.C.H., and Lam, K.N. (2020). POCUS in COVID-19: pearls and pitfalls. Lancet Respir. Med. *8*, e34.

82. Arntfield, R., et al. (2021). Development of a convolutional neural network to differentiate among the etiology of similar appearing pathological B lines on lung ultrasound: a deep learning study. BMJ open *11*, e045120.

83. Liu, L., et al. (2020). Semi-supervised active learning for COVID-19 lung ultrasound multi-symptom classification. In IEEE 32nd International Conference on Tools with Artificial Intelligence (ICTAI) (IEEE), p. 2020.

84. Borakati, A., et al. (2020). Diagnostic accuracy of X-ray versus CT in COVID-19: a propensity-matched database study. BMJ open *10*, e042946.

85. Gibbons, R.C., et al. (2020). Lung ultrasound versus chest X-ray for the diagnosis of COVID-19 pneumonia. Ann. Emerg. Med. *76*, S3.

86. Lomoro, P., et al. (2020). COVID-19 pneumonia manifestations at the admission on chest ultrasound, radiographs, and CT: single-center study and comprehensive radiologic literature review. Eur. J. Radiol. open *7*, 100231.

87. Reyes, M., et al. (2020). On the interpretability of artificial intelligence in radiology: challenges and opportunities. Radiol. Artif. Intell. *2*, e190043.

88. Selvaraju, R.R., et al. (2017). Grad-cam: visual explanations from deep networks via gradient-based localization. Proc. IEEE Int. Conf. Comput. Vis. 618–626.

89. Springenberg, J.T., Dosovitskiy, A., Brox, T., and Riedmiller, M.A. (2015). Striving for Simplicity: The All Convolutional Net. In ICLR (Workshop).

90. Gal, Y., and Ghahramani, Z. (2016). Dropout as a bayesian approximation: representing model uncertainty in deep learning. In International Conference on Machine Learning (PMLR), pp. 1050–1059.

91. Nair, T., Precup, D., Arnold, D.L., and Arbel, T. (2020). Exploring uncertainty measures in deep networks for multiple sclerosis lesion detection and segmentation. Med. image Anal. *59*, 101557.

92. Ayhan, M.S., and Berens, P. (2018). Test-time data augmentation for estimation of heteroscedastic aleatoric uncertainty in deep neural networks. In International Conference on Medical Imaging with Deep Learning. https://openreview.net/pdf?id=rJZz-knjz%20.

93. Byrne, R.M.J. (2019). Counterfactuals in explainable artificial intelligence (XAI): evidence from human reasoning. IJCAI, 6276–6282.

94. Thiagarajan, J.J., et al. (2020). Improving reliability of clinical models using prediction calibration. In Uncertainty for Safe Utilization of Machine Learning in Medical Imaging, and Graphs in Biomedical Image Analysis, C.H. Sudre, H. Fehri, T. Arbel, C.F. Baumgartner, A. Dalca, R. Tanno, K. Van Leemput, W.M. Wells, A. Sotiras, and B. Papiez, et al., eds. (Springer), pp. 71–80.

95. Nagendran, M., et al. (2020). Artificial intelligence versus clinicians: systematic review of design, reporting standards, and claims of deep learning studies. BMJ, 368. https://doi.org/10.1136/bmj.m689.

96. Kelly, C.J., et al. (2019). Key challenges for delivering clinical impact with artificial intelligence. BMC Med. *17*. https://doi.org/10.1186/s12916-019-1426-2.

97. Schaffter, T., et al. (2020). Evaluation of combined artificial intelligence and radiologist assessment to interpret screening mammograms. JAMA Netw. open *3*, e200265.

98. Organization for Economic Cooperation and Development (2021). OECD Science, Technology and Innovation Outlook 2021. https://www.oecd.org/sti/oecd-science-technology-and-innovation-outlook-25186167.htm%20.

99. Cai, X., Fry, C.V., and Wagner, C.S. (2021). International collaboration during the COVID-19 crisis: autumn 2020 developments. Scientometrics, 3683–3692.

100. Moher, D., et al. (2009). Preferred reporting items for systematic reviews and meta-analyses: the PRISMA statement. Plos Med. *6*, e1000097.

Patterns, Volume *2*

Supplemental information

On the role of artificial intelligence in medical imaging of COVID-19

Jannis Born, David Beymer, Deepta Rajan, Adam Coy, Vandana V. Mukherjee, Matteo Manica, Prasanth Prasanna, Deddeh Ballah, Michal Guindy, Dorith Shaham, Pallav L. Shah, Emmanouil Karteris, Jan L. Robertus, Maria Gabrani, and Michal Rosen-Zvi

Appendices

Appendix A. Details on meta-review

Workflow of manual review.

Fig. 1A (main manuscript) shows how the set of 465 papers for manual review was created. First, a search of the keywords "AI + Medical imaging + COVID-19" revealed 827 matches across the 4 servers *PubMed, arXiv, biorXiv and medRxiv*. Those papers were not specific to lung imaging and thus, a second set of keyword searches were made using "AI + Lung + Imaging + COVID-19 + Modality" where Modality was from {CT, X-Ray, Ultrasound}. This revealed 629 publications (visualized in Fig. 2B). Duplicates were removed and subsequently, the titles were scanned manually and papers that only touched peripherally on AI (or one of the other aspects) were removed. This led to a set of 524 papers. From the 524 manually reviewed papers, 61 were excluded during in-depth analysis since they did not involve any work on AI, leading to the final set of 463 papers.

The manual review was split across 7 authors of this paper (AC, DB, DR, EK, JB, MG, VM) and every paper was evaluated by: primary location of authors, primary location of COVID-19 data, imaging modality, task performed, data origin (external or internal) and maturity assessment.

Software for keyword search.

The number of publications per keyword were fetched via a Python package that can be used to reproduce the figures and is publicly available at: https://pypi.org/project/paperscraper/. The queries to the APIs of *PubMed*, *arXiv*, *bioRxiv* and *medRxiv* were made using synonyms or each keyword where a paper was considered a match when title or abstract contained at least one of the synonyms for each keyword (see Table A1). The reference date for all calculations was 31.12.2020.

Table A1 – List of considered synonyms per search keyword.

Keyword	Synonyms
**COVID-19**	**SARS-CoV-2, corona**
**Imaging**	**Image, screen, screening, scan**
**Medical imaging**	**Medical image**
**AI**	**Artificial intelligence, deep learning, machine learning, neural network, computer vision**
**Lung**	**Chest, pulmonary**
**Breast**	**Mammography**
**CT**	**Computed tomography**
**X-Ray**	**XRay, CXR, radiography**
**Ultrasound**	**Sonography, LUS**

Distributions of modality, task and maturity

Figure A1 provides details from the meta-review and quantifies how task and maturity are distributed by modality. For example, 87% of all 230 CXR works performed diagnosis whereas this was only the case for 58% of CT papers, where a richer set of papers was found (~15% of works on segmentation and severity assessment, whereas this was 3-4% for CXR). Projects using X-Ray for diagnosis were on average of much lower quality (82% low quality) compared to CT (60%).

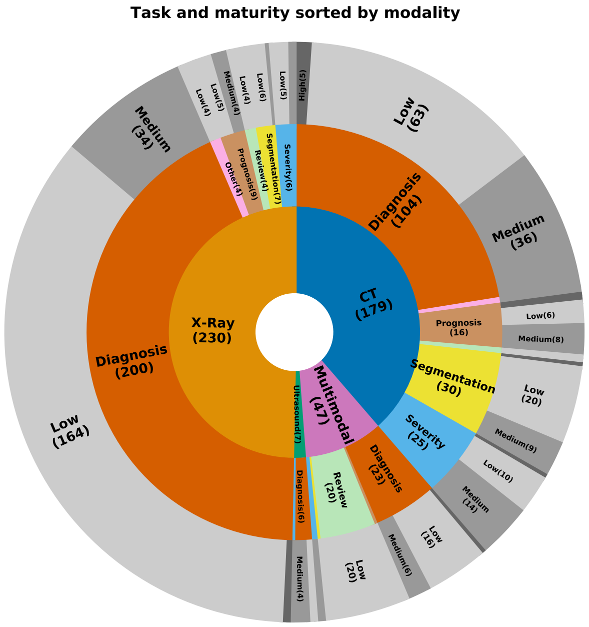

Fig. A1. Sunburst plot on the quality of AI papers distributed by task and modality. The root is in the center and layers are added hierarchically towards the periphery. Hence, the plot should be read inside-out. E.g., From the 179 papers on CT imaging, 104 were dedicated to diagnosis with 63 having low, 36 middle and 5 high maturity. Labels are omitted for fields with less than 5 publications.

Appendix B. Imaging market sizes

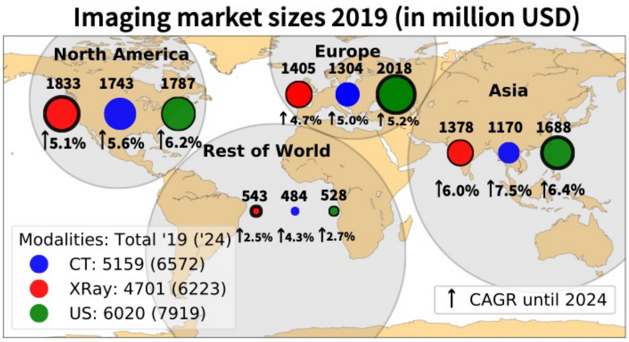

Fig. A2: Regional sizes of medical imaging markets. Overall, US has the largest market across all modalities and is especially dominant in Europe. CT instead is dominant in North America, but CAGR for US are higher and a turn-around is predicted until 2024. The circle radius is proportional to the market size, but for visual clarity, thickness of black borders resembles the regional rank per modality. Figure was created with data from https://www.marketsandmarkets.com/, Diagnostic Imaging Market – Forecast to 2024.

Appendix C. Scheme for maturity assessment

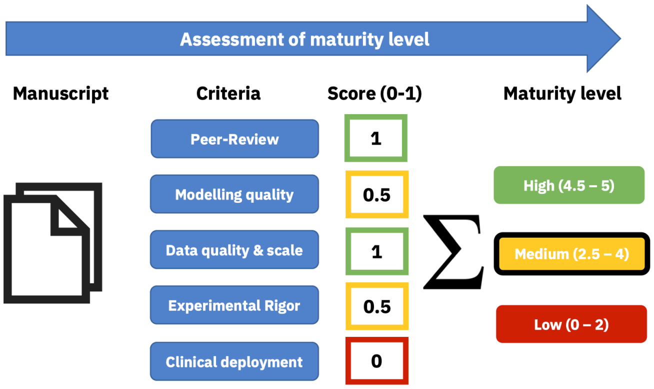

Fig. A3. Workflow for the assessment of maturity of each paper. Each paper was assigned a score for each of the five categories. For details on the individual categories, please see the text.

Criteria

Peer review

Whether or not the article appeared in a peer-reviewed journal or in Conference Proceedings. No distinction between quality of journals/conferences. This is binary score.

Examples:

**0**	Manuscript was not peer-reviewed and is preprint-only (arXiv, bioRxiv, medRxiv)
**1**	Manuscript appeared in a journal or in conference proceedings

Modeling quality

This is an umbrella category for everything related to the AI modelling approach. Mostly, this evaluates how complex/sophisticated the **methods** section of the manuscript is. Did the authors address a single task (e.g., detection) or multiple? Did the authors use off-the-shelf methods or is there clear scientific novelty? Did they devise something specific to their problem? How complex is the problem (e.g., did they only consider imaging data or also clinical tabular data).
The second important factor here is **performance**, i.e., model generalisation (how well does it work?). This should be considered in light of the experimental rigor category. This is a ternary score.

Examples:

**0**	A pre-trained CNN was finetuned on COVID-19 data
**0**	Authors used a proprietary AI software instead of writing their own code.
**0.5**	Some scientific novelty is present but not enough to warrant a "1". E.g., A small tweak was done to an existing architecture.
**0.5**	Little to no methodological novelty, but the achieved performance is high, the study seems sound.
**1**	A novel and tailored technique was devised to deal with a common problem in imaging and is shown to perform well; ideally on different datasets
**1**	Authors combined several modules for separate problems in their data (e.g., a COVID-19 detection module, a severity assessment module and a module to process clinical features).

Data quality and scale

Quality: The most important sign for high data quality is to have clinical data (as opposed to public). Secondary factors are to have multi-hospital, multi-national, multi-imaging (or generally, multimodal) data.
Scale: How much data is used for training and evaluation. Were multiple datasets used? This is a ternary score.

Examples:

**0**	Only public databases are utilized.
**0**	Authors present a dataset collected from web sources
**0.5**	Authors use several (>3) large public databases and made a significant effort to combine them in a meaningful way.
**0.5**	Authors use public databases as well as clinical data released by other publications.
**1**	Authors present a single-center dataset with >200 patients.

Experimental rigor

This refers to the number of experiments conducted in the Results section. How much results are presented which show different analysis? Did the authors perform significance tests and cross-validation? How about ablation studies? Did the evaluate on multiple, independent datasets? This is a ternary score.
Examples:

**0**	Authors reported performances from single (or repeated) runs on a single data split
**0.5**	Anything that falls in-between of the 0 and 1 descriptions
**1**	Authors compared many different architectures on different datasets and performed cross validation while reporting test data results.
**1**	Authors have few model flavors and single-split but compare them rigorously with statistical tests and ideally even radiologists.

Clinical deployment

Mature AI solution should be deployed in clinical practice or at least evaluated for their prospective clinical applicability in a realistic setting. We decide to be lenient in our interpretation of *clinical deployment.* It describes to what extent the methodology is integrated into (or was evaluated in) a clinical workflow. This is a ternary score.

Examples:

**0**	No mentioning of deployment or comparison with human experts
**0.5**	AI model deployed, but not clinical: Authors built a freely available webservice.
**0.5**	A small human-in-the-loop studies was conducted where human experts interact/compete with the model
**1**	A complex comparison study between the AI and human experts was done
**1**	The AI model was deployed and used in clinical workflow, e.g., to support decision making.
